# The prognostic significance of pretreatment systemic immune-inflammation index and prognostic nutritional index in cancer patients treated with immune checkpoint inhibitors: a systematic review and meta-analysis

**DOI:** 10.1186/s12885-026-16411-6

**Published:** 2026-06-27

**Authors:** Xiaoli Yang, Peng Fu, Hui Liu

**Affiliations:** 1 Department of Nephrology, Shidong Hospital, Yangpu District, Shanghai, Shidong Hospital Affiliated to University of Shanghai for Science and Technology 200438 China; 2 Department of Hepatobiliary Surgery and Transplantation, Key Laboratory of Carcinogenesis and Cancer Invasion of Ministry of Education, Zhongshan Hospital, Liver Cancer Institute, Fudan University, 180 Fenglin Road, Shanghai, 200032 China

**Keywords:** Systemic immune-inflammation index, Prognostic nutritional index, Prognosis, Immune checkpoint inhibitors, Cancer, Meta-analysis

## Abstract

**Background:**

Systemic immune-inflammation index (SII) and Prognostic nutritional index (PNI) are emerging inflammation-related indicators that have previously been linked to prognosis of cancer patients, but the results have been inconsistent. This meta-analysis aims to review and report the latest data regarding the prognostic value of the SII and PNI in cancer patients treated with immune checkpoint inhibitors (ICIs).

**Methods:**

We searched the relevant literature in PubMed, Cochrane Library, and Embase databases from inception to February 15, 2026. Meta-analyses were performed for overall survival (OS) and progression-free survival (PFS) to assess the prognostic role of SII and PNI in cancer patients treated with ICIs. The pooled OS and PFS were estimated using hazard ratios (HR) with 95% confidence intervals (95% CI) by the random-effects model. Subgroup analysis was also conducted to investigate the correlation between indicators and study features.

**Results:**

A total of 27 studies comprising 4000 patients treated with ICIs were included in this meta-analysis. In terms of the results based on multivariate analysis, the pooled results revealed that high SII level was an unfavorable predictor for OS (HR = 2.42, 95% CI = 1.88–3.10) and PFS (HR = 1.99, 95% CI = 1.36–2.90). In addition, lower level of PNI was correlated with worse OS (HR = 2.28, 95% CI = 1.68–3.08), as well as PFS (HR = 1.84, 95% CI = 1.56–2.17). Furthermore, subgroup analysis was conducted, and we found similar conclusions.

**Conclusion:**

Our findings support SII and PNI as promising biomarkers for adequately studied cancer types, but extending them to all malignancies requires prospective validation in less-studied cancers.

**Supplementary Information:**

The online version contains supplementary material available at https://doi.org/10.1186/s12885-026-16411-6.

## Introduction

Immunosuppression in the tumor microenvironment (TME) is one of the inducers that promote tumor cells to evade recognition and elimination through the immune system, which is also the most extensively studied mechanism to date [1]. The first mechanism of tumor-induced immunosuppression involves the accumulation of immunosuppressive cells around the tumor and the production of immunosuppressive factors. Inducing the expression of immunosuppressive molecules or their receptors can block the activation of effector T cells, which is another approach of immunosuppression [2]. The most widely described immunosuppressive molecules are programmed cell death 1 (PD-1), programmed cell death receptor-1 ligand (PD-L1), and cytotoxic T lymphocyte-associated protein-4 (CTLA-4). Immune checkpoint inhibition is an important direction for immunotherapy to eliminate immunosuppression and restore immune system function [3, 4].

Immune checkpoint inhibitors (ICIs) have delivered a shift in the treatment of cancer. They have been approved for use as single agents or in combination with chemotherapies as first or second-line therapy in various cancer types [4, 5]. ICI-based combination therapy has been a revolutionary milestone in cancer management [3]. However, only a subset of patients benefit from ICIs. The rest are unresponsive to treatment or develop resistance subsequently [4, 6].

Recent work has done much to identify the target patients who might benefit most from ICIs. Among the most recognized predictors are tumor mutational burden (TMB), microsatellite instability (MSI), and PD-L1 expression. Use of these biomarkers is hampered by a high degree of invasiveness or limited accessibility [7]. Thus, better biomarkers are urgently needed. Recently, inflammation-based prognostic biomarkers have shown excellent predictive efficiency for ICIs. However, the predictive value of inflammatory markers warrants further exploration.

The systemic immune-inflammation index (SII) is a cost-effective index calculated with the following formula: platelet count ⋅ (neutrophil count/lymphocyte count) [8]. According to previous studies, SII could be a reliable candidate in predicting long-term survival outcomes in patients with solid tumors, such as hepatocellular carcinoma (HCC), non-small-cell lung cancer (NSCLC), renal cell carcinoma (RCC), and gastric cancer (GC) [9, 12]. In contrast, there is a lack of established evidence-based medical research to clarify the applicability of SII in evaluating the clinical outcomes of patients treated with ICIs.

Malnutrition is a negative factor in clinical outcomes of cancer patients, including a decline in nutritional status, as well as poorer survival [13]. Prognostic nutritional index (PNI) is an indicator of nutritional status and systemic inflammation, calculated according to the serum albumin level and peripheral blood lymphocyte count (PNI = 10 ⋅ serum albumin + 0.005 ⋅ peripheral lymphocytes) [14]. It is a simple approach to stratify the risk of postoperative complications through assessing perioperative nutritional status and immunological status [15]. PNI is applied as a prognostic indicator in cancer patients. However, the prognostic value for patients treated with ICIs is still inconclusive [16, 18].

Hence, we conducted this comprehensive meta-analysis based on 27 studies to investigate the prognostic value of SII and PNI in patients who received ICIs. Pooled hazard ratio (HR) with 95% confidence interval (95% CI) of overall survival (OS) and progression-free survival (PFS) was used to investigate the prognostic role of SII and PNI in patients receiving ICI treatment.

## Materials and methods

### Search strategy

This meta-analysis was performed according to the guidance of the Preferred Reporting Items for Systematic reviews and Meta-Analyses (PRISMA) guidelines (Supplementary Table 1) [19], and the inclusion criteria were established according to the Population, Intervention, Comparison, Outcome, Study design (PICOS) model. Two authors (X.L.Y. and H.L.) independently performed the literature search from databases including PubMed, Cochrane Library literature, and Embase databases from the inception of the databases up to February 15, 2026. The search was restricted to studies published in English. The following terms were employed to search the literature, including “neoplasm”, “cancer”, “immune checkpoint inhibitors”, “PD-1”, “PD-L1”, “CTLA-4”, “nivolumab”, “atezolizumab”, “penpulimab”, “toripalimab”, “ipilimumab”, “Camrelizumab”, “pembrolizumab”, “tremelimumab”, “avelumab”, “immunotherapy”, “systemic immune inflammation index”, “SII”, “prognostic nutritional index”, “PNI”. The detailed search strategy is described in Supplementary Table 2.

### Inclusion and exclusion criteria

Two reviewers (X.L.Y. and H.L.) selected the literature independently. The inconsistent studies were reviewed by another researcher (P.F.) and resolved through consensus. Inclusion criteria were as follows: (1) all patients were diagnosed with specific cancer; (2) all patients were treated with ICIs; (3) studies investigated the correlation between baseline SII/PNI and survival outcome (OS, PFS); (4) published in English; (5) HRs with 95% CIs for OS and PFS were available to extract directly. For studies based on same population, the study containing the largest sample size and the most comprehensive information was included. The exclusion criteria were: (1) reviews, letters, case reports, or comments; (2) non-ICI treatment; (3) ICIs in combination with other treatments; (4) the prognostic value of SII/PNI was not investigated; (5) studies with insufficient data for analysis; (6) overlapped patients.

### Data extraction and quality assessment

Two investigators (X.L.Y. and H.L.) evaluated all potential studies independently. Thw following items were recorded from each eligible study: the first author, published year, country, cancer type, ICIs, cut-off value of baseline SII and PNI, sample size, clinical features of patients, follow-up time, survival outcomes (OS, PFS) and HR with 95% CI extracted from univariate analysis or multivariate analysis. We applied the Newcastle-Ottawa Scale (NOS) criteria for quality assessment as the studies included were cohort studies [20]. The NOS evaluates the study consisting of three aspects: selection (0–4 points), comparability (0–2 points), and outcome (0–3 points). The NOS scores range from 0 to 9, a study with NOS scores ≥ 6 was considered high quality.

### Statistical analysis

The HR and its 95% CI in univariate and multivariate analysis were extracted directly from each eligible study. Pooled HRs and 95% CIs were used to evaluate the correlation between pretreatment SII/PNI level and prognosis of cancer patients who underwent ICI treatment. Cochran Q test and the inconsistency index (I^2^) statistics were used to evaluate the heterogeneity of pooled statistics. *P* > 0.1 and I² < 50% were considered to indicate lack of heterogeneity between included studies. The random-effects model was chosen in this meta-analysis since there exists inherent clinical heterogeneity between included studies [21]. We performed sensitivity analysis to explore and identify the heterogeneity between different publications by excluding a single study. Subgroup analyses were further investigated based on region, cancer type, sample size, and cut-off value to determine the source of heterogeneity. Egger’s regression asymmetry and Begg’s rank correlation test were used to assess publication bias. *P* < 0.05 was considered statistically significant. All statistical analyses were conducted with Stata 14.0 statistical software (College Station, Texas, 77845, USA).

## Results

### Literature selection and study characteristics

**Fig. 1:**
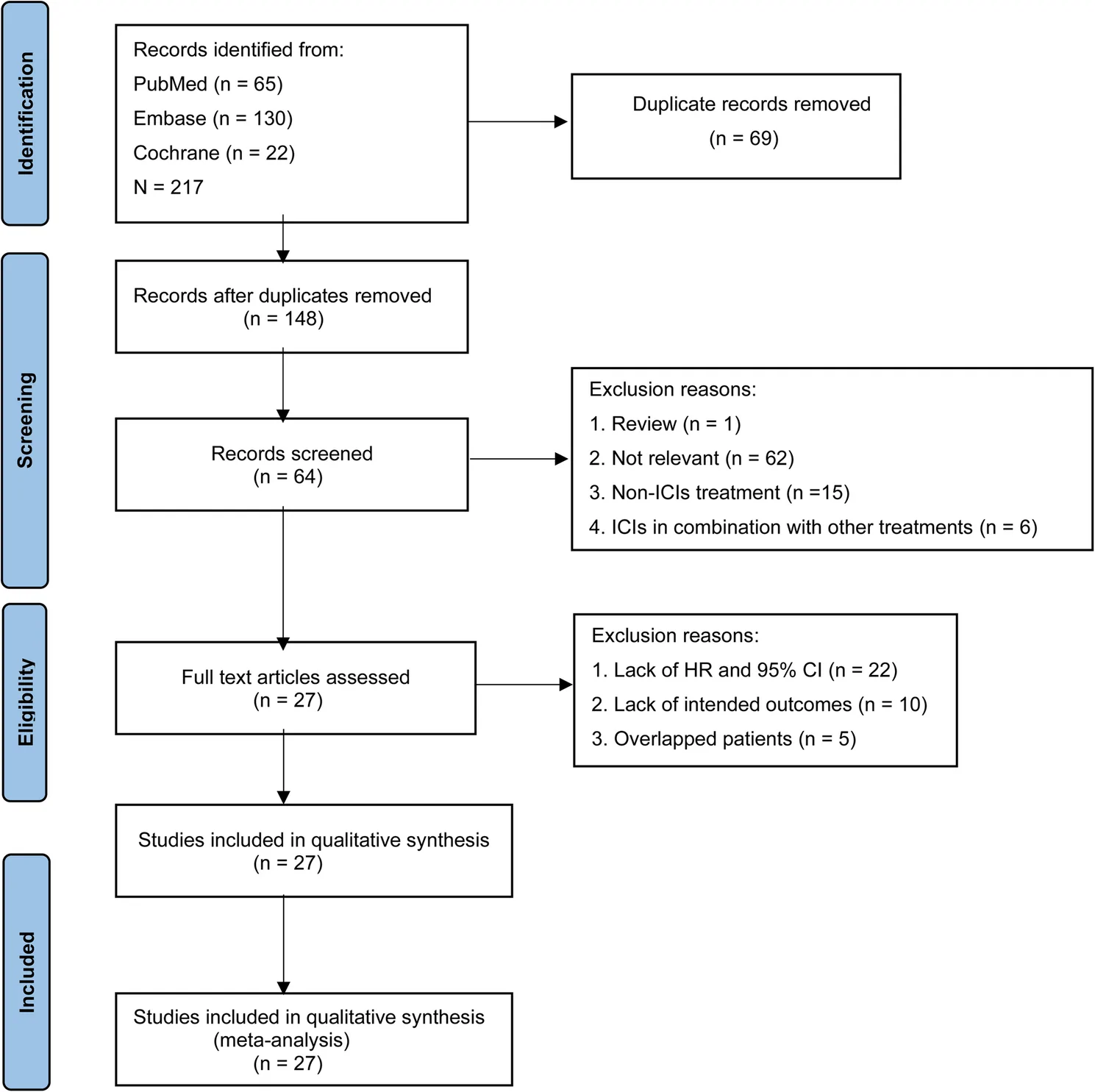
Flow chart of included studies for this meta-analysis

The initial literature search resulted in two hundred seventeen records according to the above search strategies. There were one hundred forty-eight studies remaining after excluding duplicates. After screening the titles and abstracts, eighty-four studies were removed. Subsequently, thirty-seven studies were eliminated after full-text reading. Finally, a total of twenty-seven studies from eight countries were included in this meta-analysis [8, 22, 31]. A flow diagram of the literature screening procedures is presented in Fig. 1. The detailed characteristics of the eligible studies were presented in Table 1. All included studies were published between 2019 and 2026. Eighteen eligible studies were conducted in Asia, four in Italy, two in Turkey, one each in France, the USA, and Israel. The sample size of included studies ranged from 20 to 571, with a total patients of four thousand. Nine studies were focused on NSCLC, four for GC, three for urothelial carcinoma (UC), two each for HCC, RCC, and esophageal squamous cell carcinoma (ESCC), one for mixed cancers, the remaining four studies for other cancers. The median cut-off values of PNI and SII were 42.6 and 800.2, respectively. All patients in each study were treated with ICIs. In all included studies, the HR with 95% CI of OS or PFS was reported directly. NOS scores of included studies ranged from six to eight. The details of NOS scores are presented in Supplementary Table 3.

**Table 1:** Baseline characteristics of included studies

Author (year)	Country	Cancer type	ICIs	Cut-off value		Sample size	Mean or median or range of age (years)	Sex, male (%)	Follow-up (months)	Survival analysis	NOS score
				SII	PNI						
Giorgi (2019)	Italy	RCC	Nivo	1375	-	313	65 (40–84)	75.1	NR	OS	7
Liu (2019)	China	NSCLC	Nivo	603.5	-	44	60 (43–74)	75.0	6.9	OS, PFS	8
Chen (2021)	China	GC	ICIs	665.3	-	139	60 (51–67)	74.1	23.8	OS, PFS	8
Shang (2021)	China	PC	Nivo, Pemb, Atezo, Ipi, Sinti	566	-	122	56 (33–85)	71.3	NR	OS, PFS	8
Wu (2021)	China	ESCC	Camre, Nivo, Pemb, Toripa, Sinti	829.37	-	119	61 (42–78)	85.7	NR	PFS	7
Xiong (2021)	China	SCLC	Nivo, Pemb, Atezo, Toripa	730	-	41	61 (42–80)	87.8	NR	PFS	7
Fornarini (2021)	Italy	UC	Atezo	1375	-	267	69 (62–74)	82.8	9.5	OS, PFS	8
Rebuzzi (2021)	Italy	RCC	Nivo	720	-	571	61 (49–73)	70.4	16.3	OS, PFS	8
Holtzman (2021)	Israel	NSCLC	Pemb	400	-	302	70 (36–97)	66.2	NR	OS	8
Seban (2021)	France	NSCLC	Pemb	1270	-	51	65 (37–86)	60.8	26.5^*^ for PFS	OS, PFS	8
Mei (2021)	China	HCC	Nivo, Pemb, Toripa, Sinti, Camre	268.8	48	442	52 (21–75)	86.4	13.7	OS	7
Shoji (2019)	Japan	NSCLC	Nivo, Pemb, Atezo	-	45.5	102	61 (42–80)	71.6	6.7	OS, PFS	8
Matsubara (2020)	Japan	NSCLC	Atezo	-	40	24	64.5 (49–82)	70.8	NR	OS	6
Namikawa (2020)	Japan	GC	Nivo	-	31.1	29	71 (49–86)	65.5	32	OS, PFS	7
Shimizu (2020)	Japan	UC	Pemb	-	45	27	73 (52–82)	85.2	7	OS, PFS	8
Peng (2020)	China	NSCLC	Pemb, Nivo, Toripa, Sinti	-	45	102	62	85.3	NR	OS, PFS	8
Mirili (2020)	Turkey	NSCLC	Nivo	-	35	20	61 (41–74)	85.0	9	OS	8
Guller (2021)	USA	HNSCC	Nivo, Pemb	-	45	99	64 (57–70)	86.9	NR	OS, PFS	8
Liu (2021)	China	NSCLC	Nivo, Pemb, Sinti, Camre, Toripa	-	46.05	123	59.9 ± 11.3	79.7	NR	OS, PFS	8
Shi (2021)	China	NSCLC	NR	-	45	32	NR	NR	12.9	OS, PFS	8
Wang (2021)	China	ESCC	NR	-	45	236	NR	NR	NR	OS, PFS	7
Watanabe (2021)	Japan	GC	Nivo	-	40	110	NR	71.8	NR	OS, PFS	8
Zaitsu (2021)	Japan	LC	Nivo, Pemb, Atezo	-	43	73	70.9 ± 9.4	71.2	NR	OS, PFS	8
Ishiyama (2021)	Japan	UC	Pemb	-	40	65	73 (65–79)	67.7	7.2	OS, PFS	8
Muhammed (2021)	Italy	HCC	Nivo, Pemb, Ipi, Ave, Atez, Durva	-	45	362	65 (58–70)	78.5	NR	OS, PFS	8
Guven (2021)	Turkey	Mixed	Nivo, Atezo, Pemb, Ipi	-	45	150	61 (18–86)	69.3	8.5	OS, PFS	8
Lee (2022)	Korea	GC	Nivo	-	40	35	55 (25–71)	54.2	NR	OS, PFS	8

*Abbreviations:* *SII *systemic immune-inflammation index, *PNI *prognostic nutrition index, *ICIs *immune checkpoint inhibitors, *GC *gastric cancer, *UC *urothelial carcinoma, *RCC *renal cell carcinoma, *NSCLC *non-small cell lung cancer, *PC *pancreatic cancer, *ESCC *esophageal squamous cell carcinoma, *SCLC *small cell lung cancer, *HCC *hepatocellular carcinoma, *HNSCC *head and neck squamous cell carcinoma, *LC *lung cancer, *Atezo *atezolizumab, *Nivo *nivolumab, *Pemb *penpulimab, *Toripa *toripalimab, *Ipi *ipilimumab, *Sinti *sintilimab, *Durva *durvalumab, *Camre *camrelizumab, *OS *overall survival, *PFS *progression -free survival, *NR *not reported, *NOS *Newcastle-Ottawa Scale

^*^Follow-up time for OS not reported

## Impact of SII on OS and PFS in cancer patients treated with ICIs

**Fig. 2:**
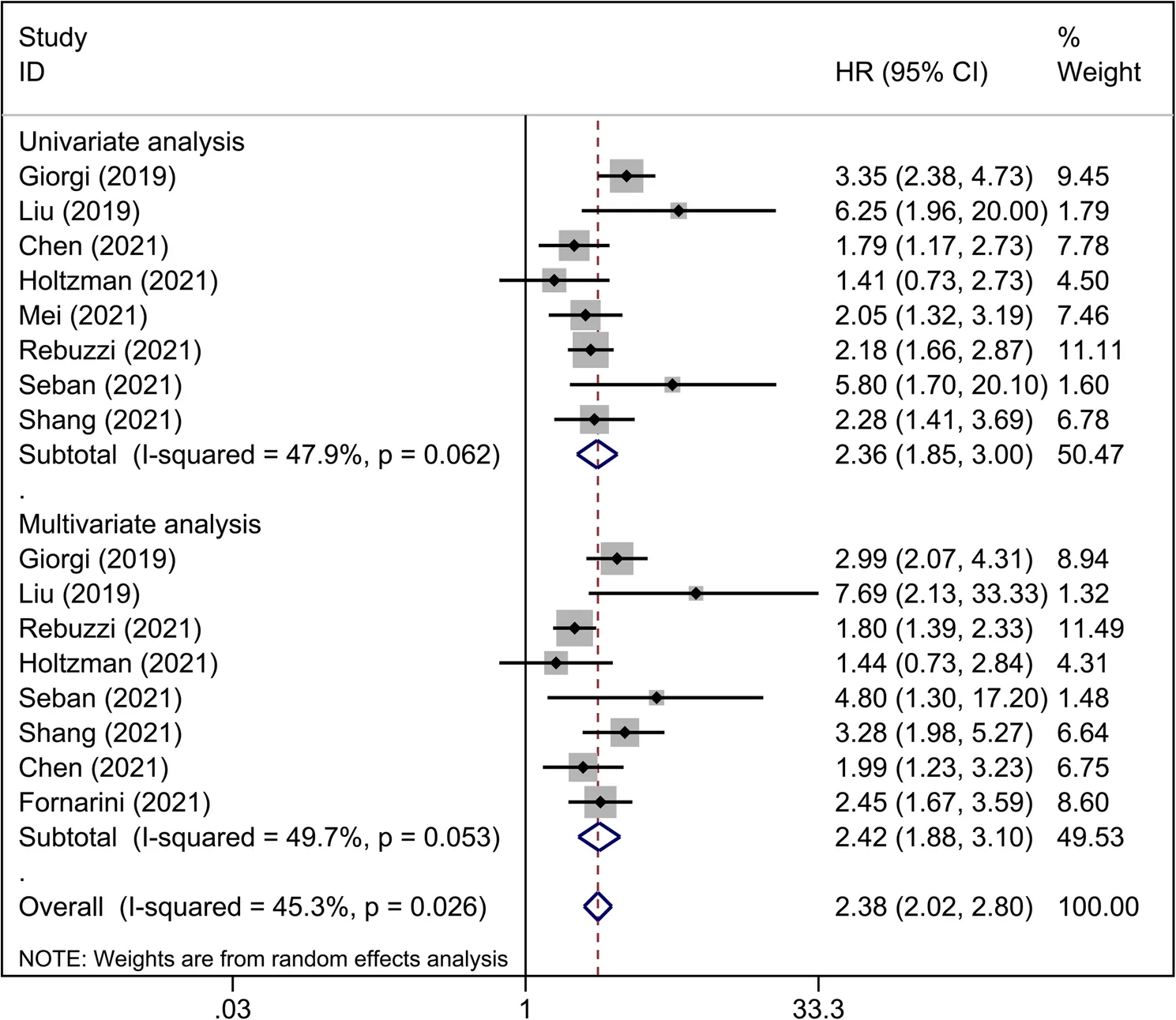
Meta-analysis of SII on OS in cancer patients treated with ICIs categorized by univariate and multivariate analysis outcomes. OS: overall survival; HR: hazard ratio; CI: confidence interval; ICIs: immune checkpoint inhibitors

**Fig. 3:**
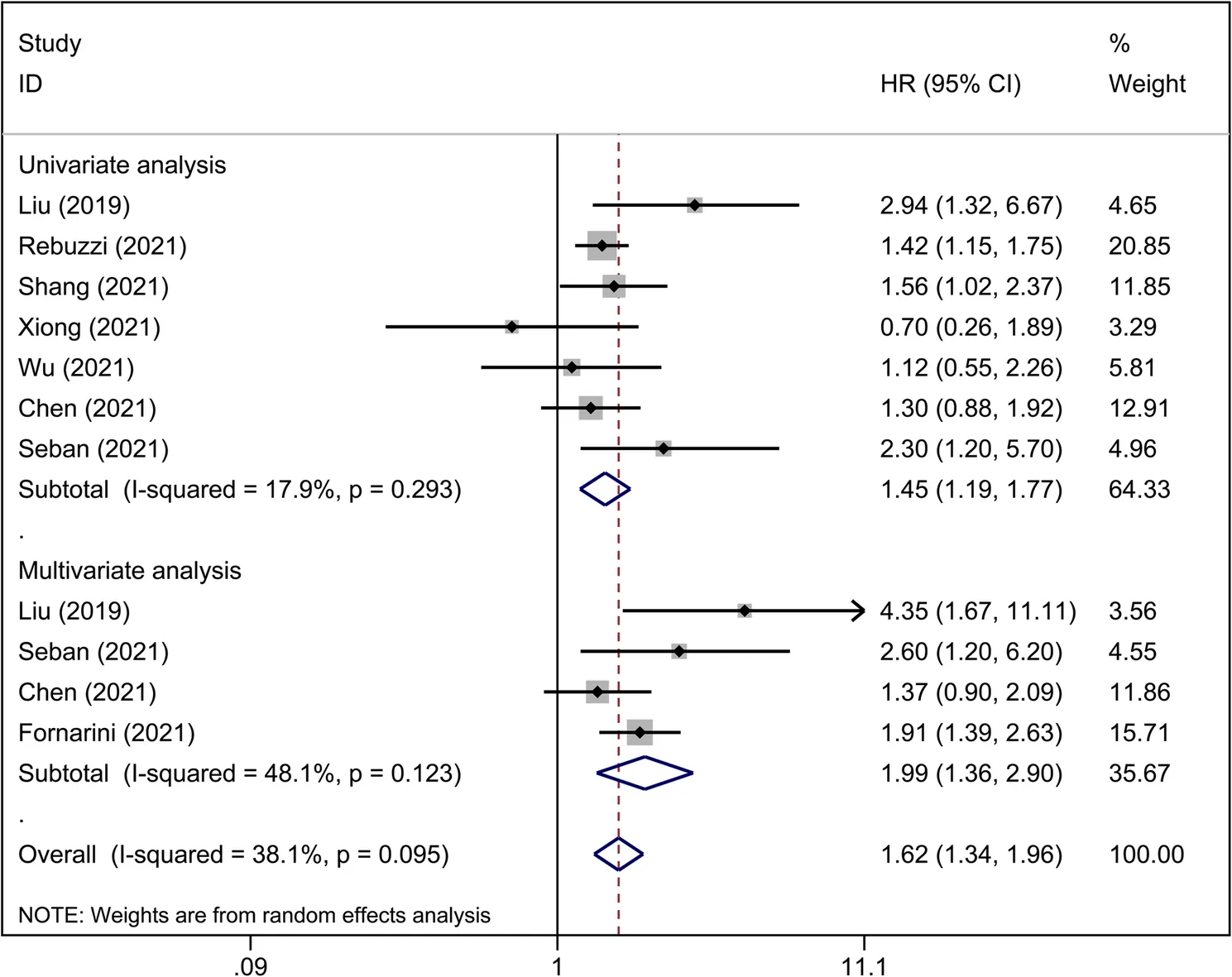
Meta-analysis of SII on PFS in cancer patients treated with ICIs categorized by univariate and multivariate analysis outcomes. PFS: progression-free survival; HR: hazard ratio; CI: confidence interval; ICIs: immune checkpoint inhibitors

As for SII, 9 of the 11 SII-related studies reported OS outcomes. The pooled results suggested high SII was significantly associated with worse OS in patients treated with ICIs (univariate analysis: HR = 2.36, 95% CI = 1.85-3.00, *P* < 0.001; multivariate analysis: HR = 2.42, 95% CI = 1.88–3.10, *P* < 0.001, Fig. 2) with a moderate level of heterogeneity (univariate analysis: I^2^ = 47.9%, *P* = 0.062; multivariate analysis: I^2^ = 49.7%, *P* = 0.053). Furthermore, the prognostic value of SII for PFS was investigated in 8 studies. Patients with high SII had inferior PFS (univariate analysis: HR = 1.45, 95% CI = 1.19–1.77, *P* < 0.001, I^2^ = 17.9%, *P* = 0.293; multivariate analysis: HR = 1.99, 95% CI = 1.36–2.90, *P* < 0.001, I^2^ = 48.1%, *P* = 0.123, Fig. 3) compared with low SII group. Notably, for SII on PFS, the pooled multivariate HR (1.99) was derived from only 4 of the 8 included studies (vs. all 8 for the univariate HR), so the two estimates reflect different study subsets rather than the same studies before and after adjustment, likely explaining the higher multivariate value (1.99) versus the univariate value (1.45). Sensitivity analysis confirmed that no individual study influenced the pooled outcomes on OS and PFS (Supplementary Figures 1 and 2).

### Impact of PNI on OS and PFS in cancer patients treated with ICIs

**Fig. 4:**
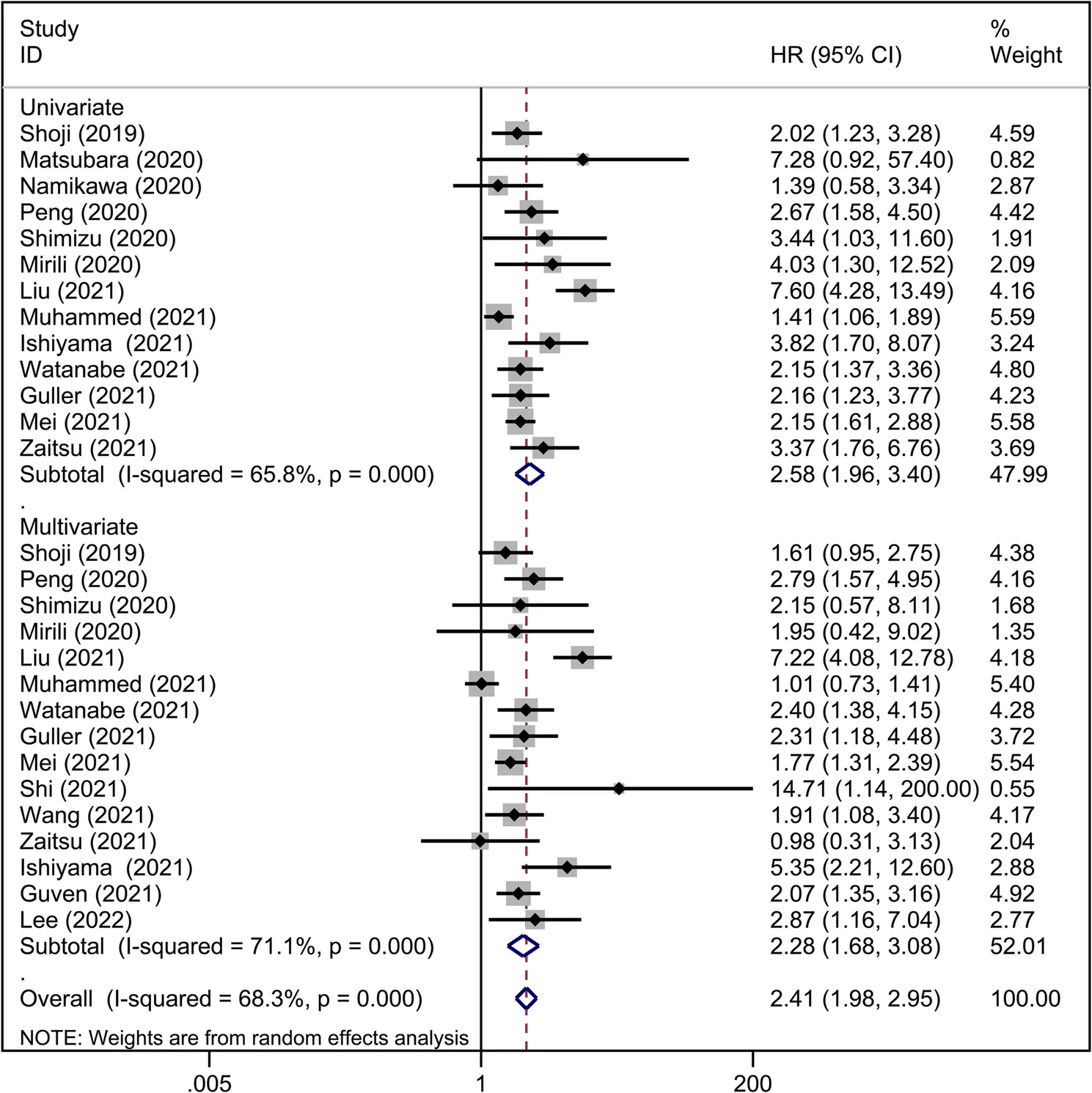
Meta-analysis of PNI on OS in cancer patients treated with ICIs categorized by univariate and multivariate analysis outcomes. OS: overall survival; HR: hazard ratio; CI: confidence interval; ICIs: immune checkpoint inhibitors

A total of 17 studies investigated the prognostic value of pretreatment PNI involving 2031 cancer patients receiving ICIs. The prognostic value for OS was accessible in all relevant studies. Pooled results indicated that patients with low PNI had significantly poorer OS (univariate analysis: HR = 2.58, 95% CI = 1.96–3.40, *P* < 0.001; multivariate analysis: HR = 2.28, 95% CI = 1.68–3.08, *P* < 0.001, Fig. 4), but significant heterogeneity existed between relevant studies (univariate analysis: I^2^ = 65.8%, *P* < 0.001; multivariate analysis: I^2^ = 71.1%, *P* < 0.001).

**Fig. 5:**
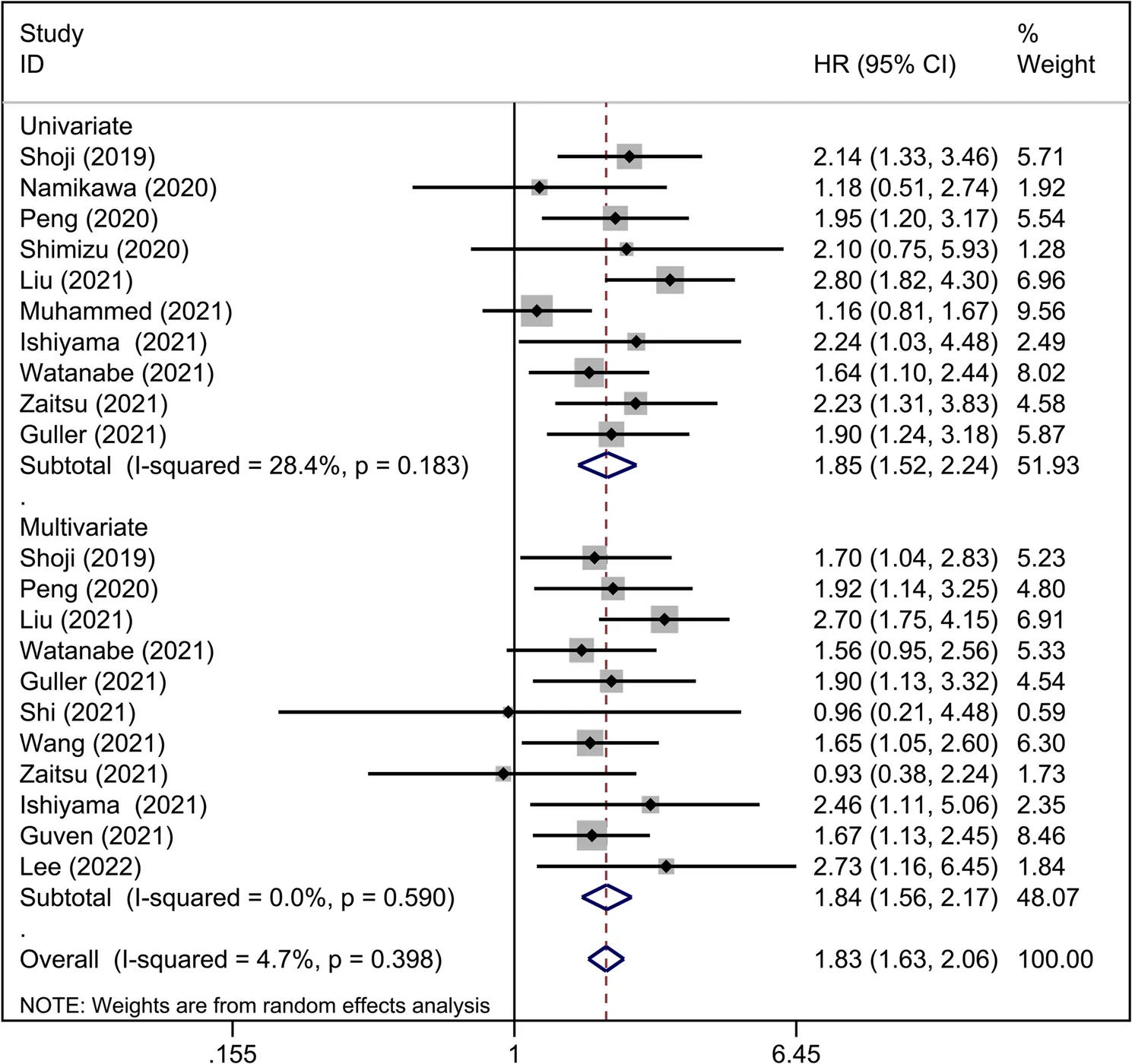
Meta-analysis of PNI on PFS in cancer patients treated with ICIs categorized by univariate and multivariate analysis outcomes. PFS: progression-free survival; HR: hazard ratio; CI: confidence interval; ICIs: immune checkpoint inhibitors

The predictive efficacy for PFS of ICI therapy was assessed in 13 studies covering 1480 patients. Pooled results revealed that low PNI was unfavorable factor for PFS (univariate analysis: HR = 1.85, 95% CI = 1.52–2.24, *P* < 0.001, I^2^ = 28.4%, *P* = 0.183; multivariate analysis: HR = 1.84, 95% CI = 1.56–2.17, *P* < 0.001, I^2^ = 0.0%, *P* = 0.590, Fig. 5). Sensitivity analysis outcomes indicated that the pooled results of OS and PFS were not significantly changed by excluding any single study (Supplementary Figs. 3 and 4).

### Subgroup analysis

We further conducted subgroup analyses to appraise the potential association between indicators and study features, focusing on country, cancer type, sample size and cut-off value. Regarding the multivariate analysis results, high SII level was associated with poorer OS (HR = 2.95, 95% CI = 1.69–5.14, *P* < 0.001), while PFS showed a decreasing trend but was not statistically significant (HR = 2.25, 95% CI = 0.73–6.91, *P* = 0.156) in Chinese patients (Table 2). Additionally, low level of PNI was consistently associated with poor OS and PFS in multivariate analysis, irrespective of cancer type, sample size, and cut-off value (Table 3).

**Table 2:** Subgroup analysis of the prognostic value for SII

	Subgroup	No. of studies	Univariate analysis HR (95% CI)	*P*	Heterogeneity		No. of studies	Multivariate analysis HR (95% CI)	*P*	Heterogeneity	
					I^2^	*P*				I^2^	*P*
OS											
Country	China	4	2.17 (1.60–2.94)	< 0.001	26.0	0.256	3	2.95 (1.69–5.14)	< 0.001	54.2	0.113
	Italy	2	2.67 (1.75–4.06)	< 0.001	72.8	0.055	3	2.31 (1.69–3.16)	< 0.001	62.3	0.070
	Others	2	2.59 (0.66–10.20)	0.715	74.5	0.048	2	2.31 (0.73–7.30)	0.155	61.8	0.106
Cancer type	NSCLC	3	3.37 (1.13–10.02)	0.029	71.7	0.029	3	3.30 (1.10–9.90)	0.033	66.8	0.049
	RCC	2	2.67 (1.75–4.06)	< 0.001	72.8	0.055	2	2.28 (1.39–3.74)	0.001	79.7	0.027
	Others	3	2.01 (1.55–2.60)	< 0.001	0.0	0.756	3	2.50 (1.93–3.24)	< 0.001	2.5	0.359
Sample size	> 100	6	2.21 (1.78–2.75)	< 0.001	40.7	0.134	6	2.27 (1.80–2.87)	< 0.001	47.5	0.090
	< 100	2	6.03 (2.59–14.06)	< 0.001	0.0	0.931	2	5.99 (2.34–15.34)	< 0.001	0.0	0.624
Cut-off value	> 700	3	2.88 (1.90–4.37)	< 0.001	62.2	0.071	4	2.40 (1.76–3.28)	< 0.001	55.1	0.083
	< 700	5	2.03 (1.53–2.70)	< 0.001	25.0	0.255	4	2.48 (1.50–4.09)	< 0.001	57.6	0.070
PFS											
Country	China	5	1.41 (1.02–1.94)	0.036	31.9	0.209	2	2.25 (0.73–6.91)	0.156	79.0	0.029
	Italy	1	1.42 (1.15–1.75)	0.001	-	-	1	1.91 (1.39–2.63)	< 0.001	-	-
	Others	1	2.30 (1.06–5.01)	0.036	-	-	1	2.60 (1.14–5.91)	0.023	-	-
Cancer type	NSCLC	**2**	2.59 (1.48–4.54)	0.001	0.0	0.669	2	3.24 (1.74–6.03)	< 0.001	0.0	0.421
	RCC	1	1.42 (1.15–1.75)	0.001	-	-	-	-	-	-	-
	Others	4	1.31 (1.01–1.69)	0.040	0.0	0.497	2	1.67 (1.21–2.30)	0.002	34.2	0.218
Sample size	> 100	4	1.40 (1.19–1.65)	< 0.001	0.0	0.851	2	1.67 (1.21–2.30)	0.002	34.2	0.218
	< 100	3	1.76 (0.79–3.92)	0.164	61.8	0.073	2	3.24 (1.74–6.03)	< 0.001	0.0	0.421
Cut-off value	> 700	4	1.37 (1.01–1.86)	0.040	21.8	0.280	2	1.99 (1.48–2.68)	< 0.001	0.0	0.493
	< 700	3	1.60 (1.11–2.29)	0.011	37.0	0.204	2	2.25 (0.73–6.91)	0.156	79.0	0.029

*Abbreviations*: *SII *systemic immune-inflammation index, *NSCLC *non-small cell lung cancer, *RCC *renal cell carcinoma, *OS *overall survival, *PFS *progression -free survival, *No*. number, *HR *hazard ratio, *95% CI *95% confidence interval

**Table 3:** Subgroup analysis of the prognostic value for PNI

	Subgroup	No. of studies	Univariate analysis HR (95%)	*P*	Heterogeneity		No. of studies	Multivariate analysis HR (95%)	*P*	Heterogeneity	
					I^2^	*P*				I^2^	*P*
OS											
Country	China	3	3.42(1.67, 6.99)	0.001	86.5	0.001	5	3.04 (1.65–5.61)	< 0.001	81.0	< 0.001
	Japan	7	2.41 (1.86–3.11)	< 0.001	0.0	0.424	5	2.18 (1.35–3.52)	0.001	45.0	0.122
	Others	3	1.90 (1.16–3.09)	0.010	54.4	0.112	4	1.74 (0.94–3.19)	0.077	64.0	0.039
Cancer type	NSCLC	5	3.67 (2.01–6.68)	< 0.001	69.9	0.010	5	3.28 (1.58–6.84)	0.001	75.2	0.003
	GC	2	1.96 (1.32–2.93)	0.001	0.0	0.385	2	2.52 (1.57–4.03)	< 0.001	0.0	0.740
	Others	6	2.24 (1.63–3.08)	< 0.001	57.0	0.040	7	1.80 (1.23–2.65)	0.003	66.1	0.007
Sample size	> 100	6	2.44 (1.65–3.61)	< 0.001	81.8	< 0.001	7	2.16 (1.40–3.35)	0.001	84.2	< 0.001
	< 100	7	2.75 (2.01–3.77)	< 0.001	0.0	0.481	7	2.64 (1.68–4.14)	< 0.001	19.8	0.279
Cut-off value	> 44	7	2.51 (1.71–3.68)	< 0.001	78.6	< 0.001	9	2.25 (1.47–3.43)	< 0.001	80.1	< 0.001
	< 44	6	2.66 (1.90–3.72)	< 0.001	12.2	0.337	5	2.56 (1.58–4.14)	< 0.001	29.1	0.228
PFS											
Country	China	2	2.38 (1.67–3.39)	< 0.001	16.3	0.274	4	2.02 (1.51–2.70)	< 0.001	12.9	0.328
	Japan	6	1.89 (1.49–2.38)	< 0.001	0.0	0.767	4	1.63 (1.21–2.20)	0.001	0.0	0.436
	Others	2	1.45 (0.90–2.35)	0.131	62.3	0.103	2	2.11 (1.33–3.32)	0.001	0.0	0.483
Cancer type	NSCLC	3	2.31 (1.77–3.01)	< 0.001	0.0	0.513	4	2.07 (1.57–2.74)	< 0.001	3.1	0.377
	GC	2	1.54(1.08–2.21)	0.018	0.0	0.488	2	1.84 (1.12–3.04)	0.017	18.4	0.268
	Others	5	1.72 (1.27–2.34)	0.001	34.0	0.194	4	1.72 (1.28–2.31)	< 0.001	0.0	0.413
Sample size	> 100	5	1.83 (1.34–2.49)	< 0.001	62.0	0.033	5	1.90 (1.53–2.35)	< 0.001	0.0	0.446
	< 100	5	1.94 (1.46–2.59)	< 0.001	0.0	0.776	5	1.83 (1.26–2.65)	0.001	8.2	0.360
Cut-off value	> 44	6	1.90 (1.42–2.53)	< 0.001	52.1	0.064	6	1.94 (1.57–2.41)	< 0.001	0.0	0.574
	< 44	4	1.80 (1.36–2.37)	< 0.001	28.4	0.183	4	1.75 (1.16–2.64)	0.007	24.3	0.266

*Abbreviations*: *PNI *prognostic nutritional index, *NSCLC *non-small cell lung cancer, *GC *gastric cancer, *OS *overall survival, *PFS *progression -free survival, *No*. number, *HR *hazard ratio, *95% CI* 95% confidence interval

### Publication bias

**Fig. 6:**
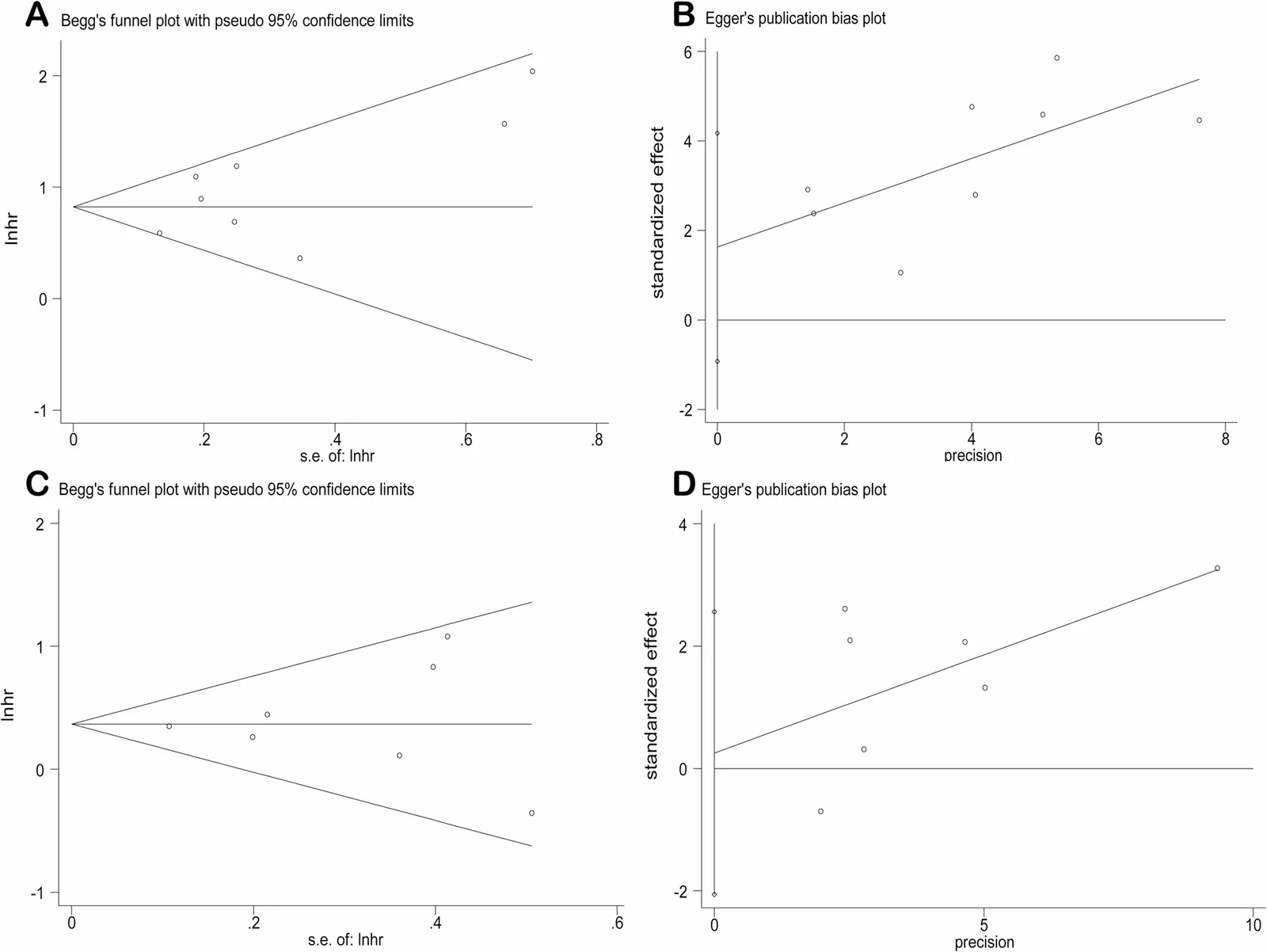
Plots for publication bias assessment of SII. **A **Begg’s test for OS in multivariate analysis, *P* = 0.386; (**B**) Egger’s test for OS in multivariate analysis, *P* = 0.170; (**C**) Begg’s test for PFS in univariate analysis, *P* = 1.000; (**D**) Egger’s test for PFS in univariate analysis, *P* = 0.789

**Fig. 7:**
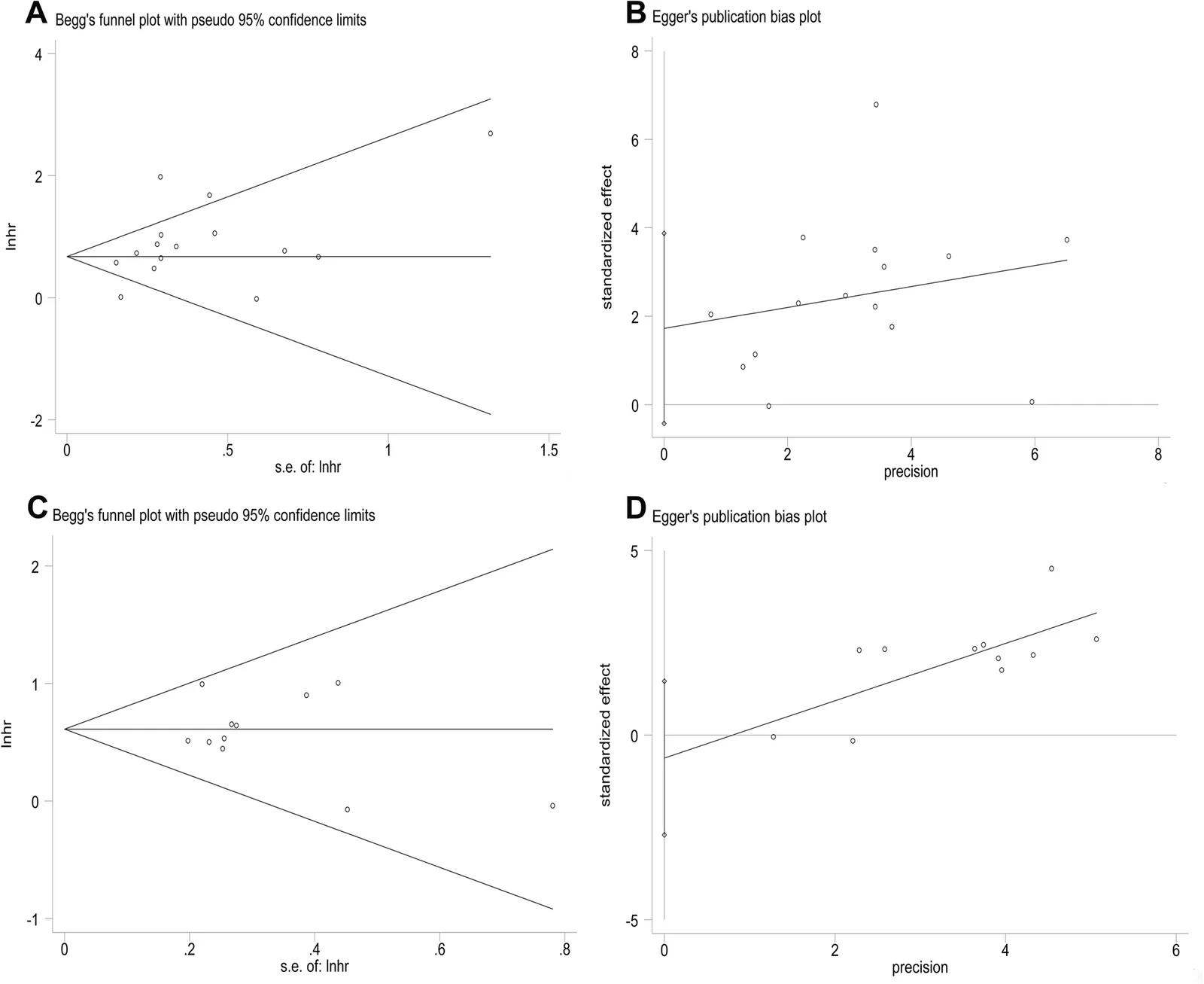
Plots for publication bias assessment of PNI in multivariate analysis. **A **Begg’s test for OS, *P* = 0.235; (**B**) Egger’s test for OS, *P* = 0.107; (**C**) Begg’s test for PFS, *P* = 1.000; (**D**) Egger’s test for PFS, *P* = 0.517

Egger’s and Begg’s tests were applied to identify the potential publication bias for univariate and multivariate analysis results. No evidence for significant publication bias was found for the impact of SII on OS (Begg’s test: *P* = 0.386; Egger’s test: *P* = 0.170, Fig. 6) in multivariate analysis. The results of Begg’s and Egger’s tests verified that there was no significant publication bias of SII on PFS (Begg’s test: P *=* 1.000; Egger’s test: *P* = 0.789) in univariate analysis. Likewise, it appears to be free of publication bias of PNI on OS (Begg’s test: *P* = 0.235; Egger’s test: *P* = 0.107) and PFS (Begg’s test: *P* = 1.000; Egger’s test: *P* = 0.517) in multivariate analysis (Fig. 7). Of note, a Begg’s test *P* value of exactly 1.000, though uncommon, can occur when the rank correlation statistic reaches its minimum given the data distribution. This does not indicate a statistical error. We verified all outputs using Stata 14.0, and the results were reproducible. Furthermore, Egger’s tests consistently showed no significant publication bias, supporting the robustness of our findings. Plots of publication bias tests for univariate analysis are presented in Supplementary Figs. 5–10. We did not test publication bias for subgroups containing fewer than 10 studies, as such analyses are considered unreliable.

## Discussion

The discovery of ICIs markedly improves survival with lasting clinical benefits even after discontinuation of treatment [5]. Instead of benefiting from ICI treatment, some patients experience accelerated progression of their cancer during ICI therapy, a phenomenon known as hyperprogressive disease (HPD). HPD was first described by Ferrara et al. in 2018 [32], referring to a paradoxical acceleration of tumor growth following immune checkpoint inhibitor administration. The reported incidence of HPD varies between 4% and 29% depending on cancer type and diagnostic criteria [33]. HPD has been observed across multiple tumor types, including non-small cell lung cancer, melanoma, and head and neck cancer. Potential risk factors include older age, certain genetic mutations, and locoregional recurrence [34]. While the underlying mechanisms remain unclear, they may involve Fc receptor-mediated macrophage reprogramming or hyperactivation of the PI3K/Akt pathway. Identifying reliable biomarkers to predict HPD is clinically urgent [35]. Therefore, identifying appropriate indicators to guide patient selection for ICI treatment is necessary. Blood-based prognostic biomarkers have shown excellent potential for predicting outcomes in ICI-treated patients, characterized by being noninvasive and clinically accessible. The prognostic efficacy of SII and PNI in predicting prognosis for patients treated with ICIs is inconclusive. The present meta-analysis demonstrated that SII has shown predictive value on OS and PFS.

Additionally, we confirmed the prognostic role of PNI in cancer patients treated with ICIs. In subgroup analysis, the unfavorable impact of PNI is independent of cancer type, sample size, and cut-off value. Therefore, SII and PNI are appropriate biomarkers to stratify the beneficiary of ICI therapy. SII was first proposed by Hu et al. as a novel index to predict the prognosis of patients with hepatocellular carcinoma after curative resection [36]. The more effective value of SII over other inflammatory biomarkers has been confirmed in several oncological studies [37, 39]. SII is calculated from peripheral neutrophil, platelet, and lymphocyte counts, thereby integrating markers of both the innate inflammatory response and the adaptive immune status, and may therefore serve as a comprehensive surrogate of the systemic immune-inflammatory balance. Beyond this systemic level, the cellular composition of the immune microenvironment within the tumor also plays a critical role in determining the response to ICIs. The immune cells located in TME include T cells, regulatory T cells (Treg cells), regulatory B cells (Bregs), natural killer cells (NK cells), dendritic cells (DCs) and macrophages [40]. Among these, tumor-infiltrating lymphocytes (TILs), particularly cytotoxic T lymphocytes (CTLs), play a central role in anti-tumor immunity [41, 42]. Metastatic melanoma with infiltration of cytotoxic T lymphocytes (CTLs) at the tumor margin indicates better response to ICIs [43]. Therefore, the presence of TILs is critical for the success of immune checkpoint inhibition. Compared to the accessibility of SII, the complex and costly measurement limits the application of TILs.

Inflammation is considered an essential hallmark of tumor progression. Moreover, inflammation contributes to the TME with bioactive molecules and immune cells [44, 45]. Under homeostatic status, the balance between pro-inflammatory and anti-inflammatory responses is regulated by immune checkpoints [46]. Negative regulation of CTLs by the PD-1/PD-L1 axis has been observed [47]. CTLs are the most important defensive weapon in TME. Of note, the prognostic significance of CTL infiltration depends on its spatial distribution and functional status. Marginal CTL presence predicts favorable ICI response, whereas massive intra-tumoral accumulation often reflects chronic antigen stimulation and subsequent exhaustion. However, the phenomenon of massive CTL infiltration is a manifestation of dysfunctional state in T cells. The highly exhausted cells have altered the expression of cytokine profiles and surface markers. Exhausted cells have a reduced capacity to induce proinflammatory cytokines and fail to kill tumor cells [40].

According to the definition of SII, possible mechanisms for the prognosis of SII could be illustrated in the following aspects: thrombocythemia, neutrophilia, and lymphopenia. Previous studies have provided evidence that neutrophils and platelets can secrete cytokines, such as vascular endothelial growth factor (VEGF), which promote the angiogenesis and survival of tumors [48, 49]. Platelet-associated chemokines have shown the capacity to modulate immune response in TME [50]. Additionally, neutrophils can block the activation of T-cells to cause immune evasion of tumor cells [51]. Given the pivotal role of lymphocytes in ICI therapy, patients with lymphopenia may not derive satisfactory benefit from ICI treatment [52].

The simplified version of PNI was proposed in 1984, which is based on serum albumin and total lymphocyte count [7]. On the one hand, lymphocyte count is an important reflection of immune response, as the decrease of lymphocytes indicates poor anti-tumor effect [7, 53]. On the other hand, serum albumin concentration represents the nutritional status of patients [24]. Serum albumin, as a negative acute-phase protein, is suppressed by pro-inflammatory cytokines such as IL-6 and TNF-α that are characteristically elevated in cancer-related systemic inflammation. Thus, hypoalbuminemia may serve as a surrogate marker of systemic inflammatory burden and cancer-associated cachexia, both of which have been linked to impaired systemic immune competence and accelerated clearance of ICI monoclonal antibodies, potentially resulting in reduced drug exposure and attenuated antitumor immune response. Compared with intratumoral metabolic competition, this systemic inflammation/cachexia-mediated pathway provides a more direct mechanistic link between serum albumin levels and ICI efficacy [54].

To our knowledge, this is the first meta-analysis to investigate the prognostic value of baseline SII and PNI in patients treated with ICIs. In this study, a comprehensive analysis was performed to explore the prognostic value of two biomarkers from dual aspects of nutritional and immune status. The two indicators provide a simple and noninvasive method for predicting the prognosis of ICI treatment based on the physical condition of patients. There are still limitations to be considered. First, there exists clinical heterogeneity as studies investigated the prognostic value of indicators in different cancer types and ICI agents. Second, most of the included studies provided meaningful results, whereas some negative outcomes might not have been published as they were deemed meaningless. Besides, only articles published in English were included in this study. These two factors may potentially contribute to the existence of publication bias. Given that 12 of the included studies had sample sizes below 100, the statistical power of these individual studies may be limited. Therefore, these results may not have sufficient validity to robustly support the findings. Furthermore, the cut-off values for SII and PNI varied considerably across studies, ranging from 268.8 to 1375 for SII and from 31.1 to 48 for PNI. This variability may contribute to clinical and statistical heterogeneity. However, the use of a random-effects model helps accommodate such heterogeneity. Importantly, subgroup analyses based on cut-off values demonstrated that the prognostic effects remained consistent across different thresholds, suggesting that the association between SII/PNI and survival is robust despite cut-off variability. Given the regional and tumor-type differences observed in our subgroup analyses, population- and cancer-specific cut-off values may further facilitate clinical translation. Nevertheless, future studies should strive to establish uniform, externally validated cut-off values to enable more reliable clinical implementation.

## Conclusion

In summary, we recommend applying SII and PNI as complementary means to predict clinical outcomes of patients treated with ICIs, as we demonstrated that high SII and low PNI levels are significantly associated with poor survival following ICI treatment. In subsequent studies, large-scale, prospective, and multicenter studies are still needed to validate their clinical value in various cancer types.

## Supplementary information

Supplementary Material 1.

## Data Availability

The data that support the findings of this study are available from the corresponding author upon reasonable request.

## Abbreviations

SII: Systemic immune-inflammation index
PNI: Prognostic nutritional index
ICIs: Immune checkpoint inhibitors
OS: Overall survival
PFS: Progression-free survival
HR: Hazard ratio
95% CI: 95% confidence interval
TME: Tumor microenvironment
PD-1: Programmed cell death-1
PD-L1: Programmed cell death ligand-1
CTLA-4: Cytotoxic T lymphocyte-associated protein-4
TMB: Tumor mutational burden
MSI: Microsatellite instability
HCC: Hepatocellular carcinoma
NSCLC: Non-small-cell lung cancer
RCC: Renal cell carcinoma
NOS: Newcastle-Ottawa Scale
HPD: Hyperprogressive disease
TILs: Tumor-infiltrating lymphocytes
CTLs: Cytotoxic T lymphocytes
PICOS: Population, Intervention, Comparison, Outcome, Study design
