# Supplementary material for: The prognostic significance of pretreatment systemic immune-inflammation index and prognostic nutritional index in cancer patients treated with immune checkpoint inhibitors: a systematic review and meta-analysis
Source: BMC Cancer. 2026 Jun 27;26:1098. doi: 10.1186/s12885-026-16411-6 (PMC13579754; doi:10.1186/s12885-026-16411-6)
Supplement: Supplementary file 1 [file 12885_2026_16411_MOESM1_ESM.pdf]

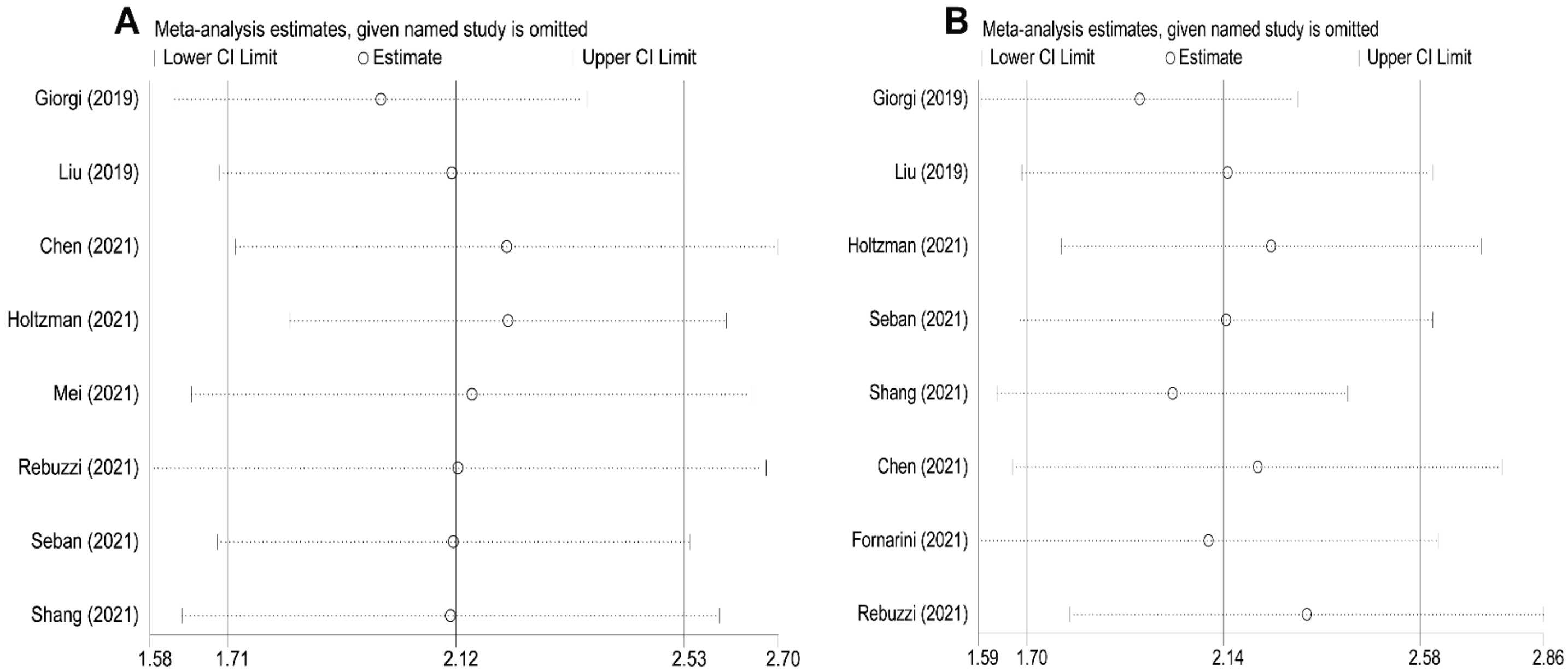

Supplementary Figure 1: Sensitivity analysis of SII on OS categorized by univariate and multivariate analysis outcomes. (A) univariate analysis; (B) multivariate analysis.

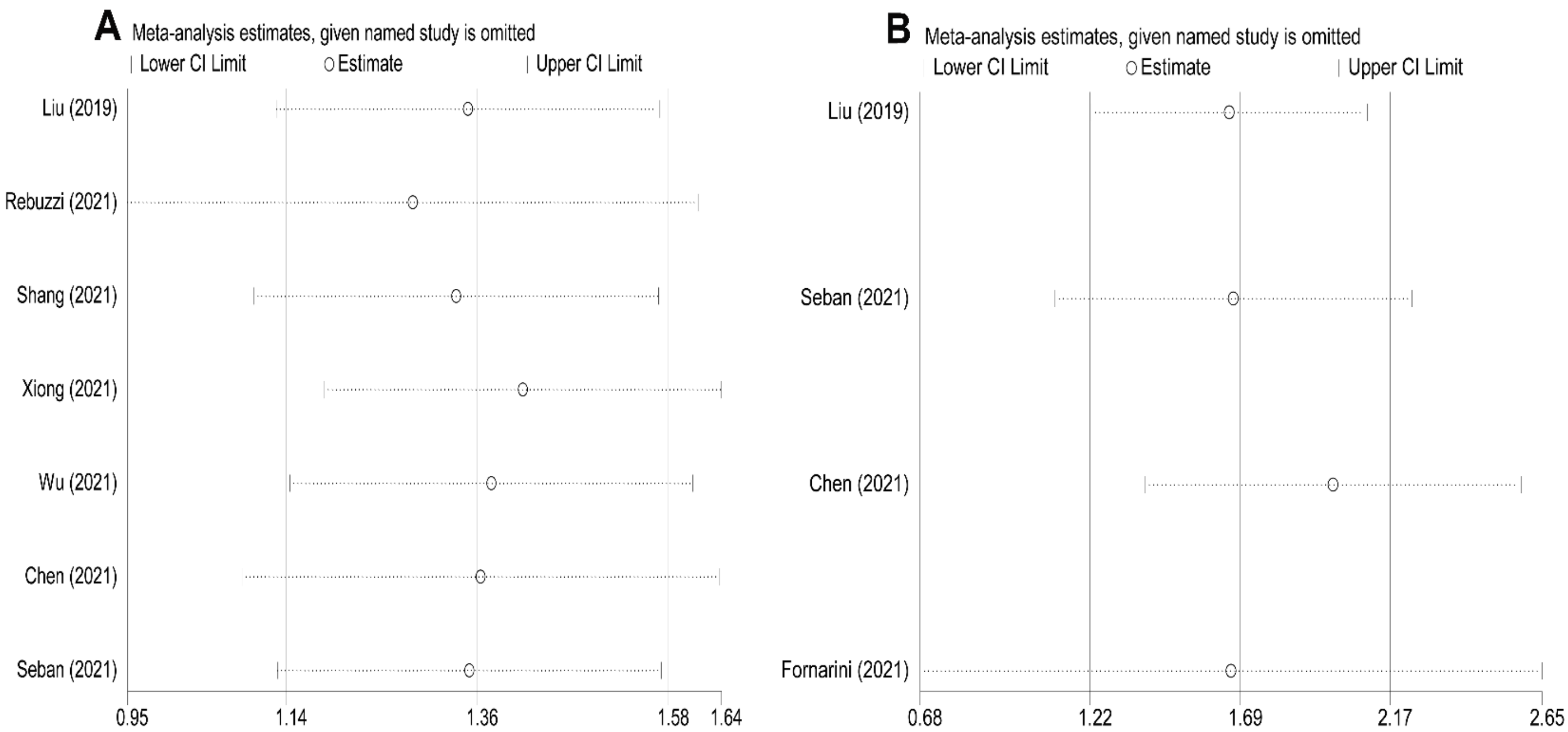

Supplementary Figure 2: Sensitivity analysis of SII on PFS categorized by univariate and multivariate analysis outcomes. (A) univariate analysis; (B) multivariate analysis.

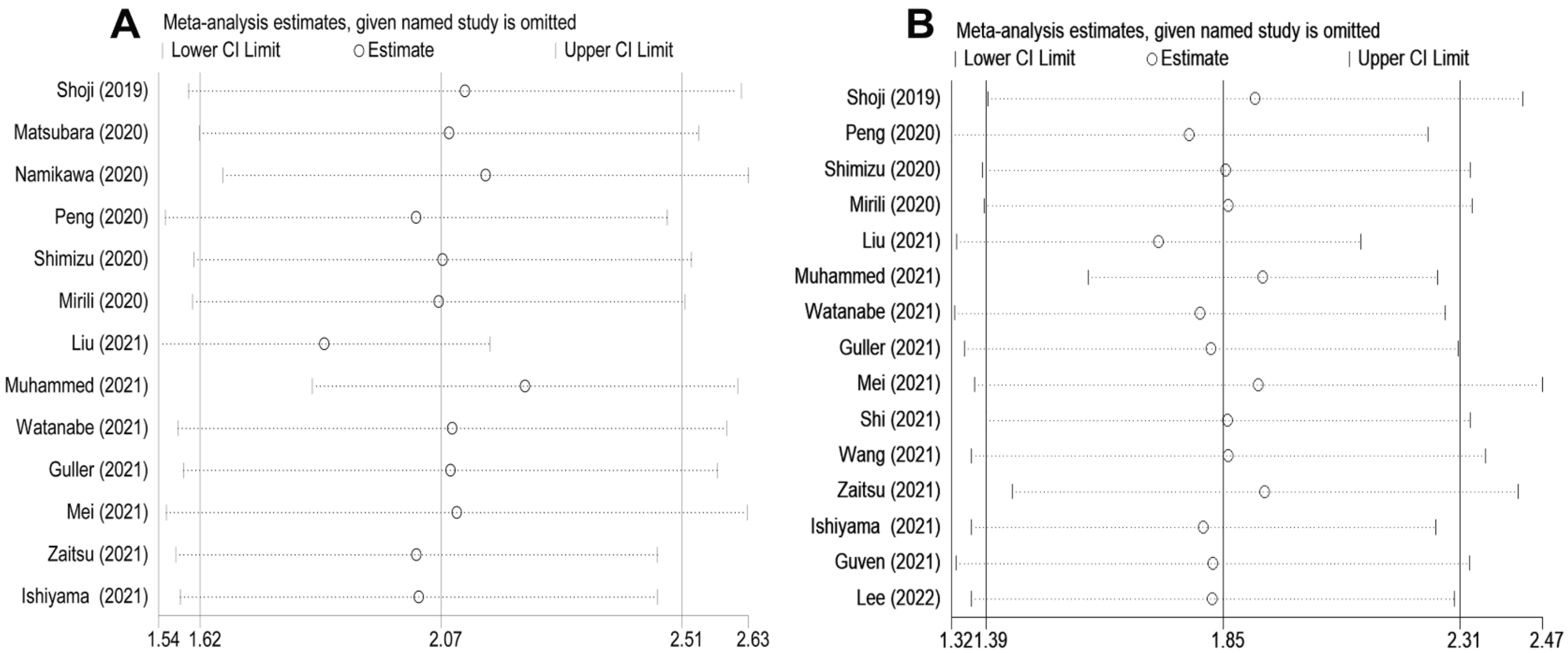

Supplementary Figure 3: Sensitivity analysis of PNI on OS categorized by univariate and multivariate analysis outcomes.

(A) univariate analysis; (B) multivariate analysis.

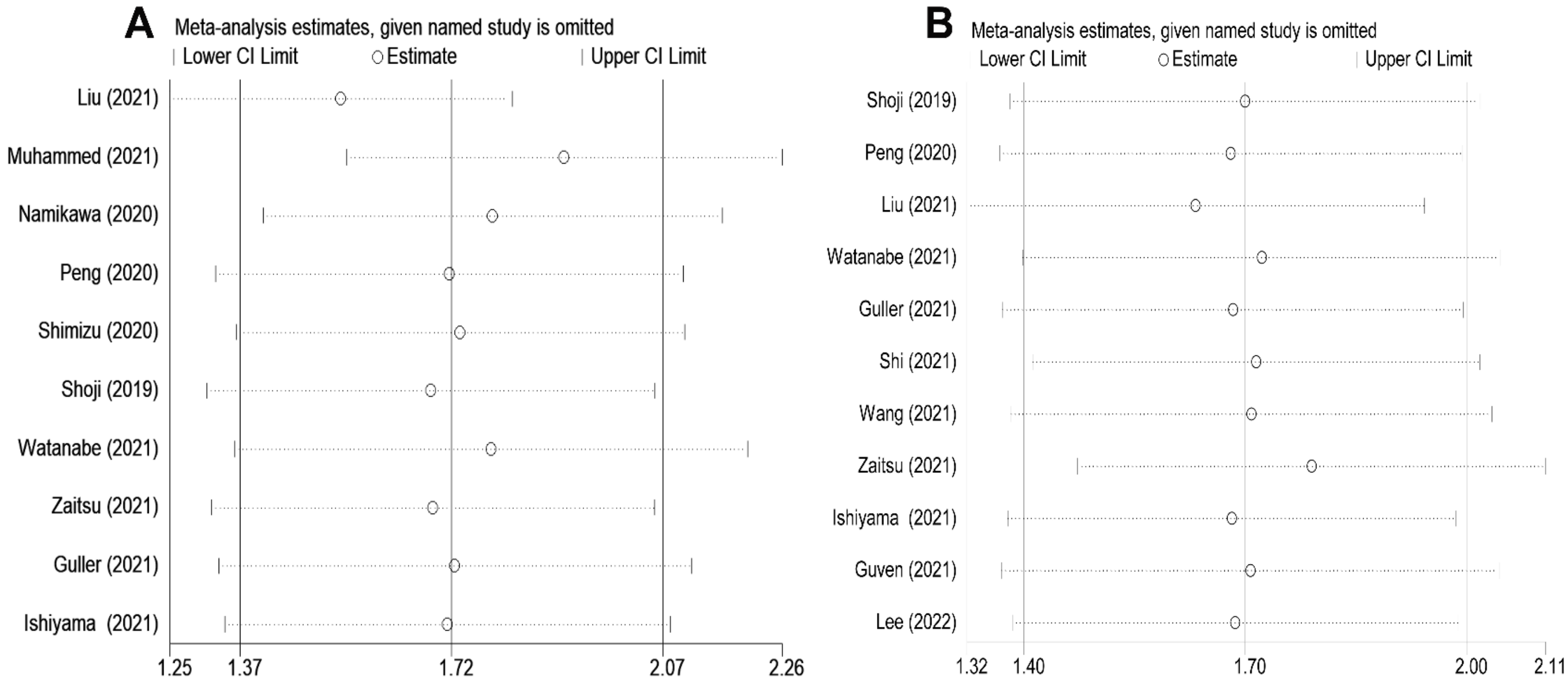

Supplementary Figure 4: Sensitivity analysis of PNI on PFS categorized by univariate and multivariate analysis outcomes.

(A) univariate analysis; (B) multivariate analysis.

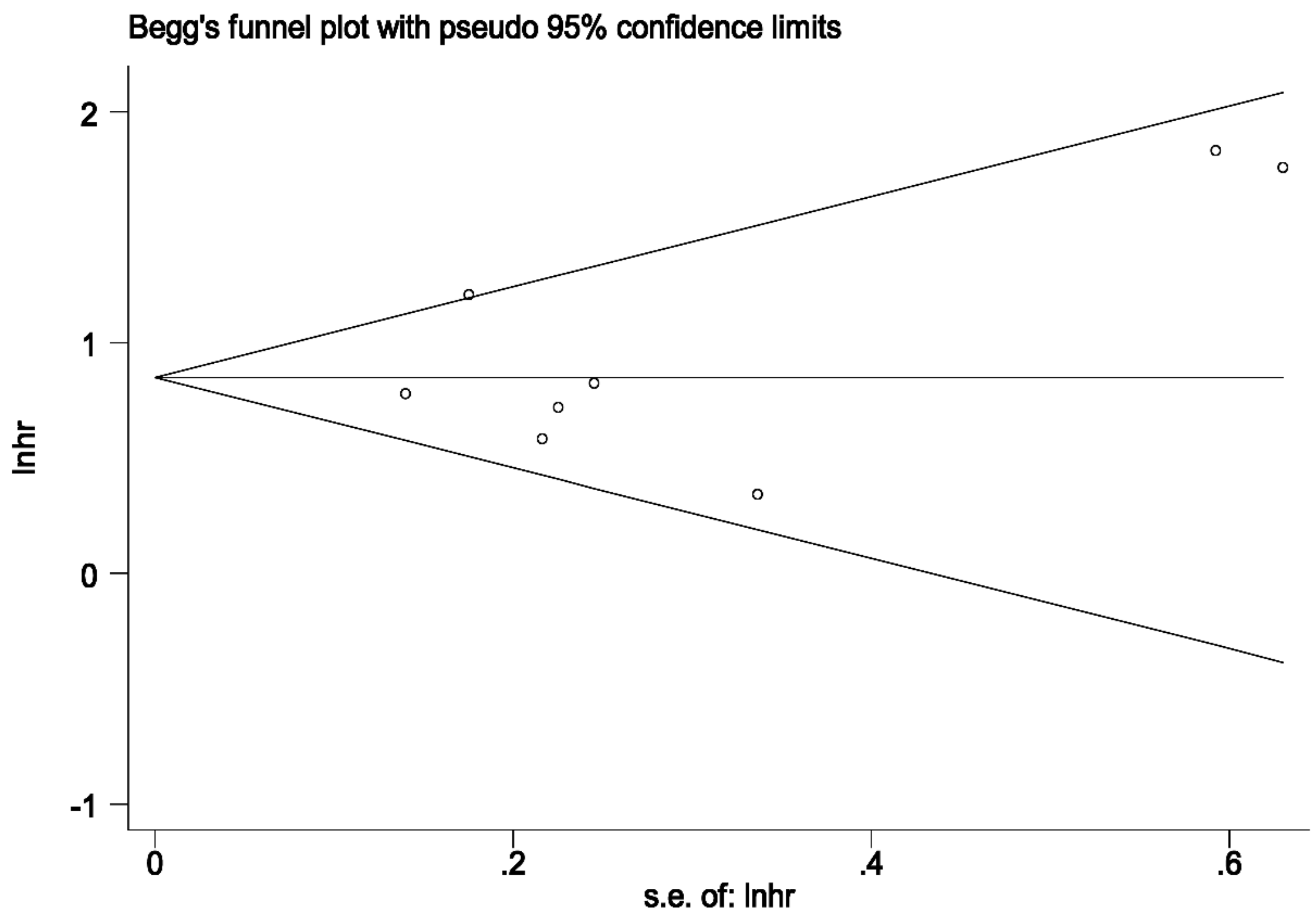

Supplementary Figure 5: Begg's test plot for publication bias assessment of SII on OS in univariate analysis, *P* = 0.902.

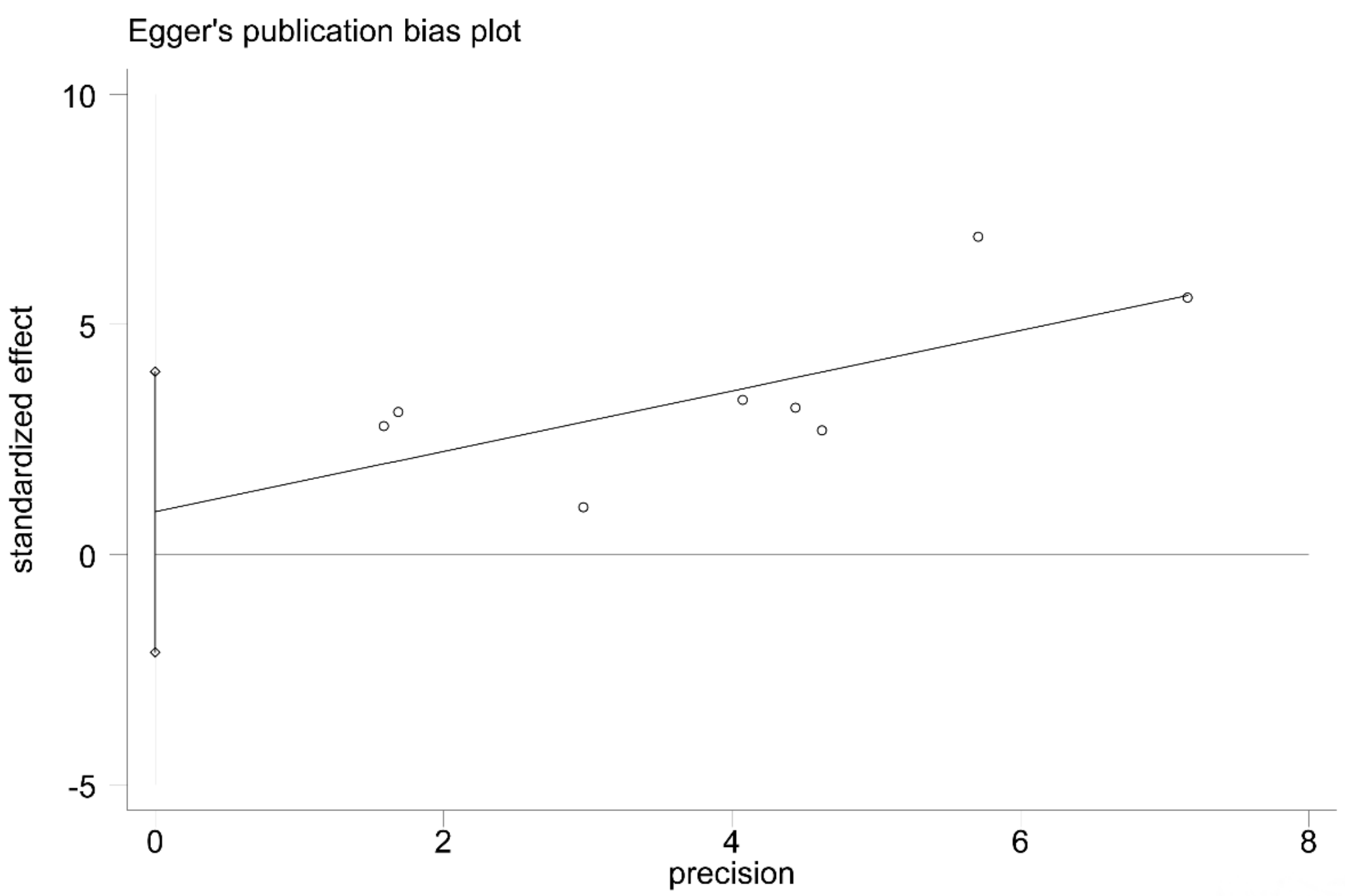

Supplementary Figure 6: Egger's test plot for publication bias assessment of SII on OS in univariate analysis， *P* = 0.485.

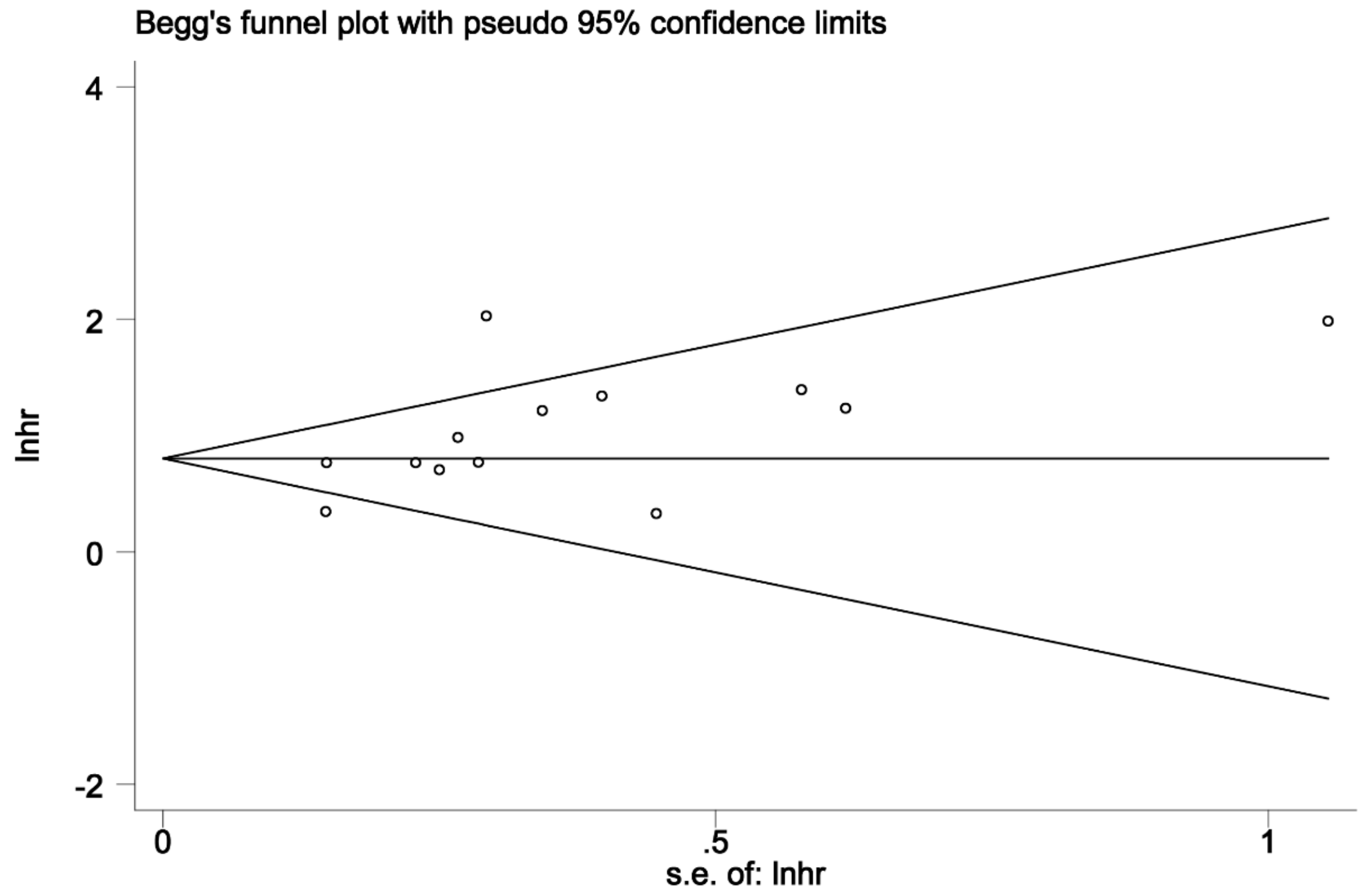

Supplementary Figure 7: Begg’s test plot for publication bias assessment of PNI on OS in univariate analysis, *P* = 0.059.

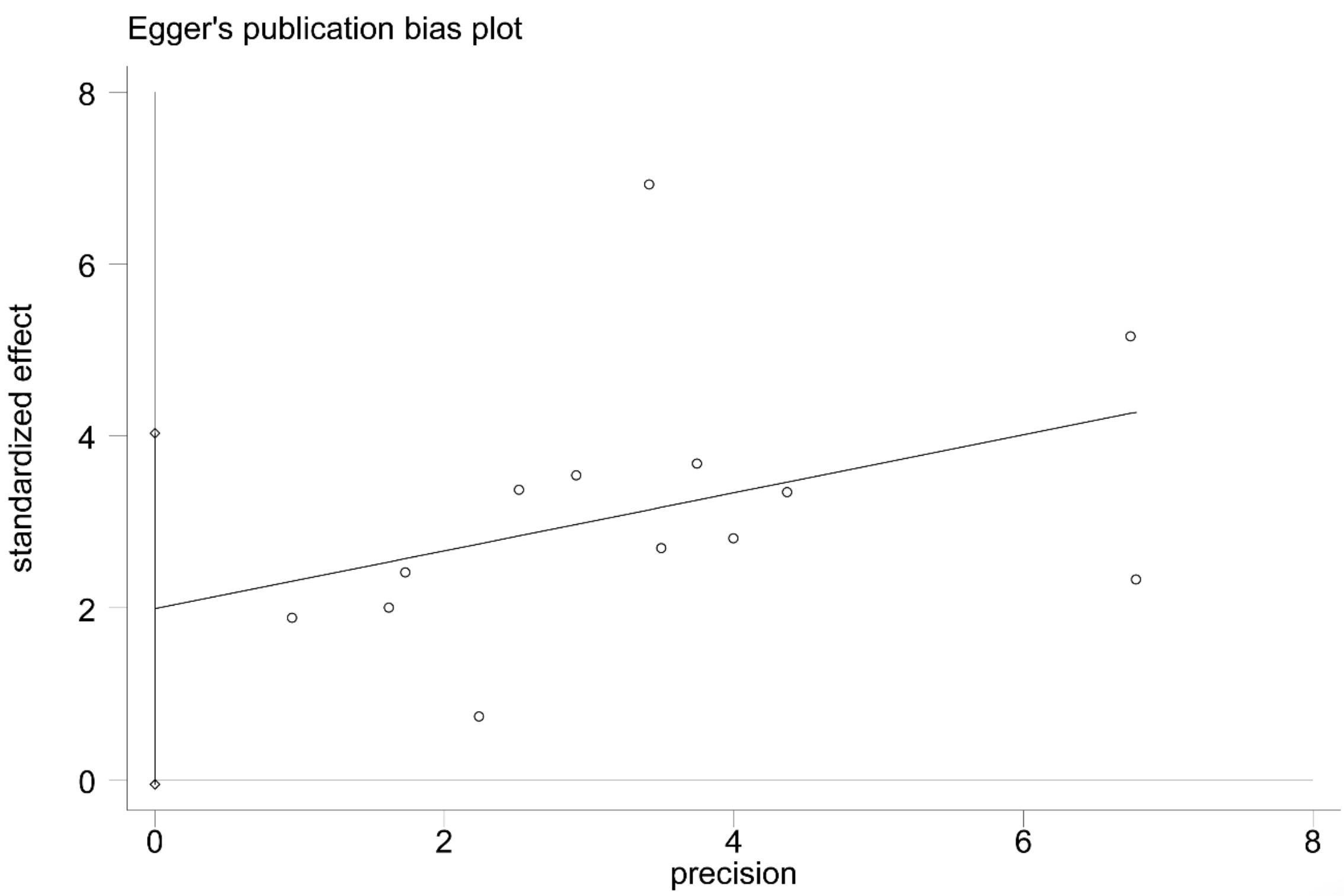

Supplementary Figure 8: Egger’s test plot for publication bias assessment of PNI on OS in univariate analysis, *P* = 0.055.

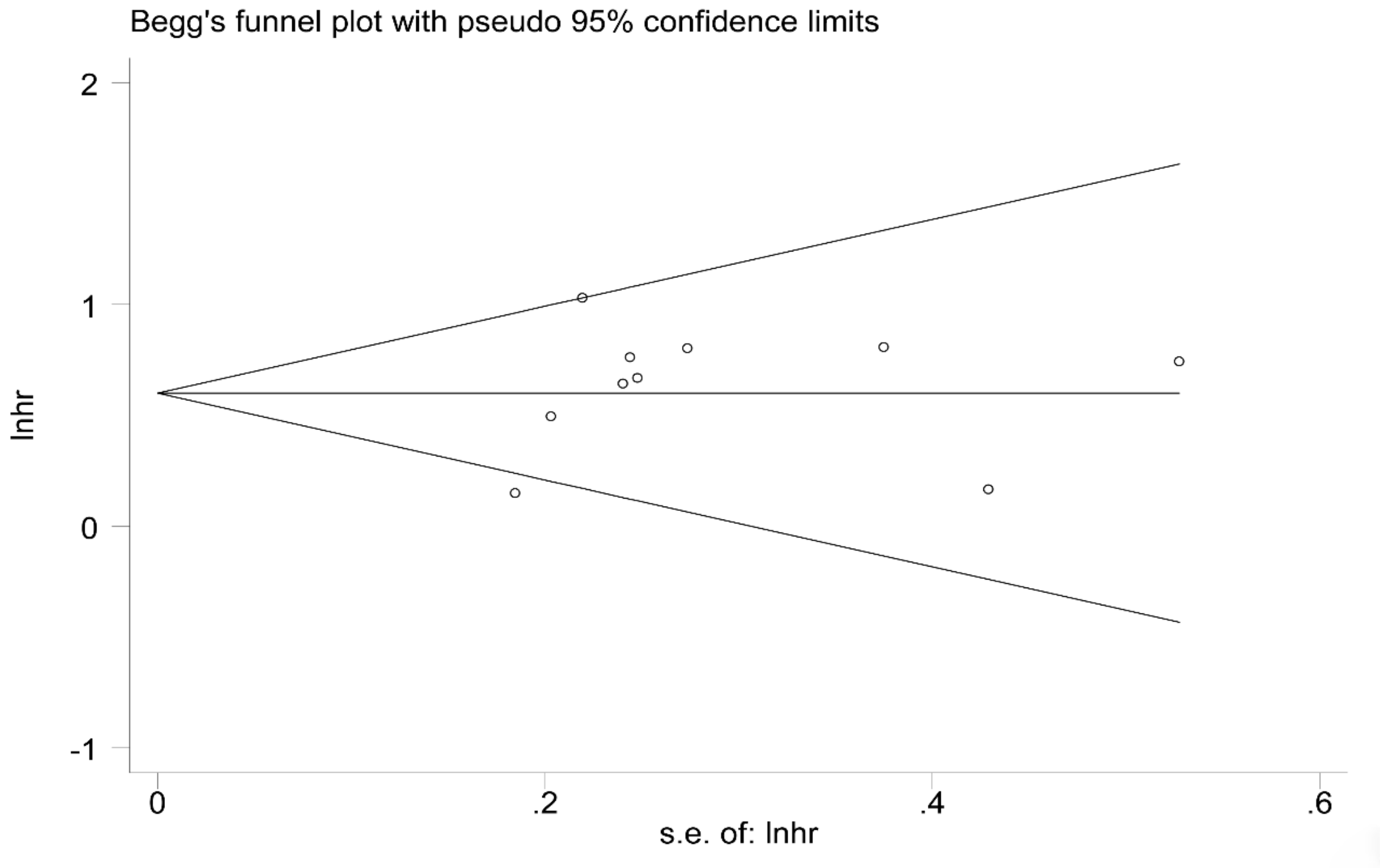

Supplementary Figure 9: Begg’s test plot for publication bias assessment of PNI on PFS in univariate analysis, *P* = 0.721.

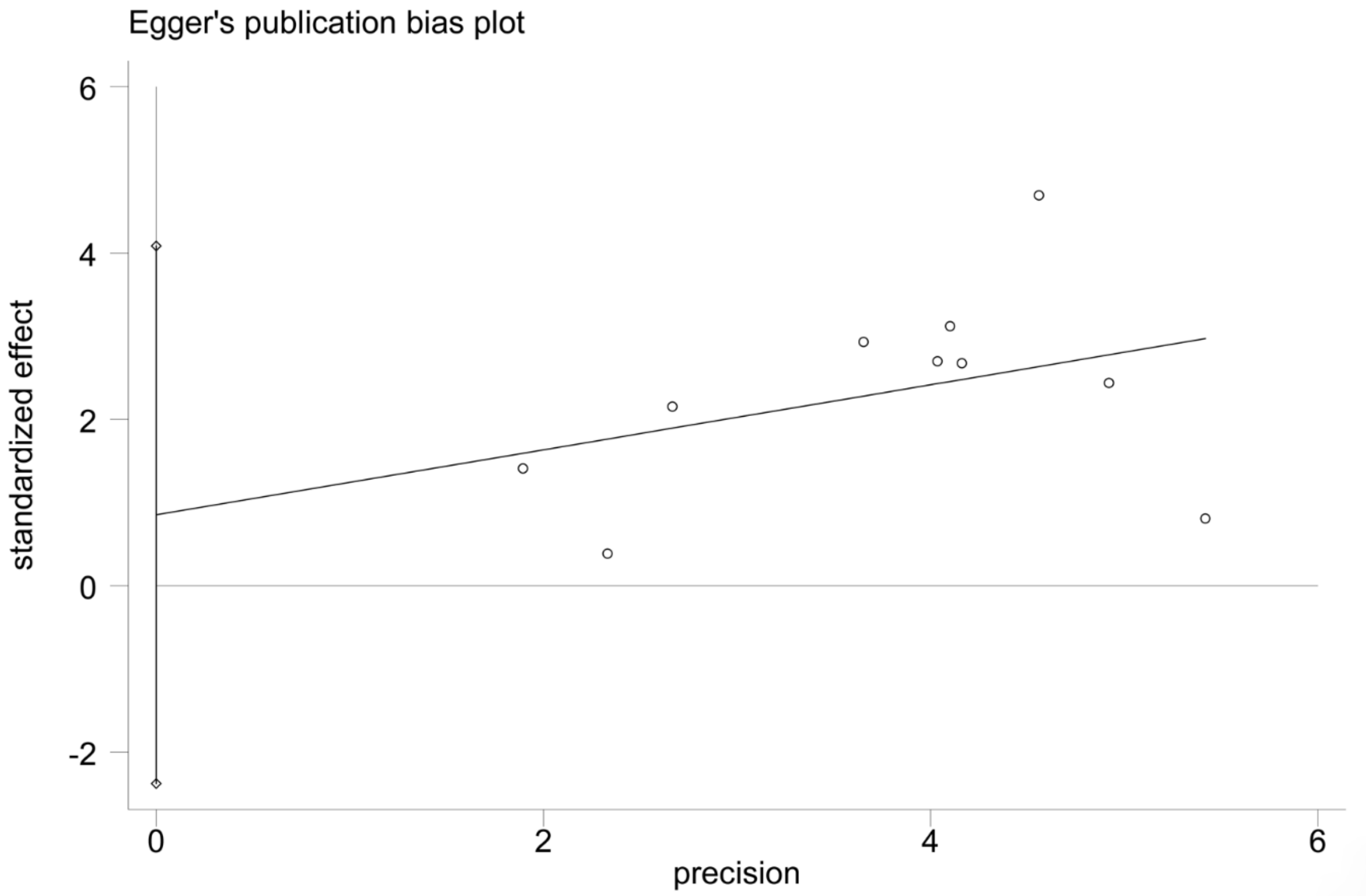

Supplementary Figure 10: Egger’s test plot for publication bias assessment of PNI on PFS in univariate analysis, *P* = 0.560.

Supplementary Table 1. Database-specific search strategy for PubMed

P (population)	Keywords searched for in abstract and title	neoplasm OR tumor OR cancer OR carcinoma OR malignant neoplasm OR benign neoplasm
	MeSH headings	"Neoplasms"[Mesh]
I (intervention)	Keywords searched for in abstract and title	immune checkpoint inhibitors OR immune checkpoint inhibition OR immune checkpoint blockers OR immune checkpoint blockade OR programmed cell death protein 1 inhibitor OR PD-1 OR programmed death ligand 1 inhibitor OR PD-L1 OR cytotoxic t lymphocyte associated protein 4 inhibitor OR CTLA-4 OR ICIs OR ICBs OR nivolumab OR atezolizumab OR penpulimab OR toripalimab OR tislelizumab OR ipilimumab OR camrelizumab OR pembrolizumab OR tremelimumab OR durvalumab OR avelumab OR immunotherapy OR immunotherapy
	MeSH headings	none
C (comparison)	Keywords searched for in abstract, title	systemic immune inflammation index OR SII OR prognostic nutritional index OR PNI
	MeSH headings	none
O (outcome)	none	
S (study design)	none	
Additional limits	Limits to English language only	

Supplementary Table 2. Details of Newcastle-Ottawa Scale

Study	Selection				Comparability	Outcome			Score
	Representativeness of the exposed cohort (1 point)	Selection of the non-exposed cohort (1 point)	Ascertainment of exposure (1 point)	Demonstration that outcome of interest was not present at start of study (1 point)	Comparability of cohorts on the basis of the design or analysis (2 point)	Assessment of outcome (1 point)	Follow-up long enough for outcomes to occur (1 point)	Adequacy of follow up of cohorts (1 point)	
Chen (2021)	1	1	1	1	1	1	1	1	8
Giorgi (2019)	1	1	1	1	1	1	1	1	8
Liu (2019)	1	1	1	1	1	1	1	1	8
Shoji (2019)	1	1	1	1	1	1	1	1	8
Peng (2020)	1	1	1	1	1	1	1	1	8
Mirili (2020)	1	1	1	1	1	1	1	1	8
Shimizu (2020)	1	1	1	1	1	1	1	1	8
Matsubara (2020)	1	1	1	1	0	1	1	1	7
Namikawa (2020）	1	1	1	1	0	1	1	1	7
Fornarini (2021)	1	1	1	1	1	1	1	1	8
Holtzman (2021）	1	1	1	1	1	1	1	1	8
Rebuzzi (2021)	1	1	1	1	1	1	1	1	8
Seban (2021)	1	1	1	1	1	1	1	1	8
Shang (2021)	1	1	1	1	1	1	1	1	8

Wu (2021)	1	1	1	1	0	1	1	1	7
Xiong (2021)	1	1	1	1	0	1	1	1	7
Mei (2021)	1	1	1	1	0	1	1	1	7
Guller (2021)	1	1	1	1	1	1	1	1	8
Liu (2021)	1	1	1	1	1	1	1	1	8
Muhammed (2021)	1	1	1	1	1	1	1	1	8
Shi (2021)	1	1	1	1	1	1	1	1	8
Wang (2021)	1	1	1	1	1	1	0	0	6
Watanabe (2021)	1	1	1	1	1	1	1	1	8
Zaitsu (2021)	1	1	1	1	1	1	1	1	8
Ishiyama (2021)	1	1	1	1	1	1	1	1	8
Guven (2021)	1	1	1	1	1	1	1	1	8
Lee (2022)	1	1	1	1	1	1	1	1	8

Supplementary Table 3. PRISMA Checklist

Section/topic	#	Checklist item	Reported on page #
**TITLE**			
Title	1	Identify the report as a systematic review and meta-analysis.	1
**ABSTRACT**			
Structured summary	2	Provide a structured summary including, as applicable: background; objectives; data sources; study eligibility criteria, participants, and interventions; study appraisal and synthesis methods; results; limitations; conclusions and implications of key findings; systematic review registration number.	2
**INTRODUCTION**			
Rationale	3	Describe the rationale for the review in the context of what is already known.	3-4
Objectives	4	Provide an explicit statement of questions being addressed with reference to participants, interventions, comparisons, outcomes, and study design (PICOS).	5
**METHODS**			
Protocol and registration	5	Indicate if a review protocol exists, if and where it can be accessed (e.g., Web address), and, if available, provide registration information including registration number.	N/A
Eligibility criteria	6	Specify study characteristics (e.g., PICOS, length of follow-up) and report characteristics (e.g., years considered, language, publication status) used as criteria for eligibility, giving rationale.	5-6
Information sources	7	Describe all information sources (e.g., databases with dates of coverage, contact with study authors to identify additional studies) in the search and date last searched.	5
Search	8	Present full electronic search strategy for at least one database, including any limits used, such that it could be repeated.	5
Study selection	9	State the process for selecting studies (i.e., screening, eligibility, included in systematic review, and, if applicable, included in the meta-analysis).	6-7
Data collection process	10	Describe method of data extraction from reports (e.g., piloted forms, independently, in duplicate) and any processes for obtaining and confirming data from investigators.	6

Data items	11	List and define all variables for which data were sought (e.g., PICOS, funding sources) and any assumptions and simplifications made.	5-6
Risk of bias in individual studies	12	Describe methods used for assessing risk of bias of individual studies (including specification of whether this was done at the study or outcome level), and how this information is to be used in any data synthesis.	6
Summary measures	13	State the principal summary measures (e.g., risk ratio, difference in means).	6
Synthesis of results	14	Describe the methods of handling data and combining results of studies, if done, including measures of consistency (e.g., I^2) for each meta-analysis.	7
**Section/topic**	**#**	**Checklist item**	**Reported on page #**
Risk of bias across studies	15	Specify any assessment of risk of bias that may affect the cumulative evidence (e.g., publication bias, selective reporting within studies).	6
Additional analyses	16	Describe methods of additional analyses (e.g., sensitivity or subgroup analyses, meta-regression), if done, indicating which were pre-specified.	7
**RESULTS**			
Study selection	17	Give numbers of studies screened, assessed for eligibility, and included in the review, with reasons for exclusions at each stage, ideally with a flow diagram.	7
Study characteristics	18	For each study, present characteristics for which data were extracted (e.g., study size, PICOS, follow-up period) and provide the citations.	7-8
Risk of bias within studies	19	Present data on risk of bias of each study and, if available, any outcome level assessment (see item 12).	7
Results of individual studies	20	For all outcomes considered (benefits or harms), present, for each study: (a) simple summary data for each intervention group (b) effect estimates and confidence intervals, ideally with a forest plot.	8-9
Synthesis of results	21	Present results of each meta-analysis done, including confidence intervals and measures of consistency.	8-9
Risk of bias across studies	22	Present results of any assessment of risk of bias across studies (see Item 15).	9
Additional analysis	23	Give results of additional analyses, if done (e.g., sensitivity or subgroup analyses, meta-regression [see Item 16]).	9
**DISCUSSION**			

Summary of evidence	24	Summarize the main findings including the strength of evidence for each main outcome; consider their relevance to key groups (e.g., healthcare providers, users, and policy makers).	10
Limitations	25	Discuss limitations at study and outcome level (e.g., risk of bias), and at review-level (e.g., incomplete retrieval of identified research, reporting bias).	12-13
Conclusions	26	Provide a general interpretation of the results in the context of other evidence, and implications for future research.	13
**FUNDING**			
Funding	27	Describe sources of funding for the systematic review and other support (e.g., supply of data), role of funders for the systematic review.	1
